# Laser powder bed fusion (LPBF) of commercially pure titanium and alloy development for the LPBF process

**DOI:** 10.3389/fbioe.2023.1260925

**Published:** 2023-09-07

**Authors:** Fabian Haase, Carsten Siemers, Joachim Rösler

**Affiliations:** Institute for Materials Science, Technische Universität Braunschweig, Braunschweig, Germany

**Keywords:** titanium, LPBF, CP titanium, alloy development, process parameter optimization, medical applications, constitutional supercooling

## Abstract

Laser powder bed fusion (LPBF) of titanium or titanium alloys allows fabrication of geometrically more complex and, possibly, individualized implants or osteosynthesis products and could thus improve the outcome of medical treatments considerably. However, insufficient LPBF process parameters can result in substantial porosity, decreasing mechanical properties and requiring post-treatment. Furthermore, texturized parts with anisotropic properties are usually obtained after LPBF processing, limiting their usage in medical applications. The present study addresses both: first, a design of experiments is used in order to establish a set of optimized process parameters and a process window for LPBF printing of small commercially pure (CP) titanium parts with minimized volume porosity. Afterward, the first results on the development of a biocompatible titanium alloy designed for LPBF processing of medical implants with improved solidification and more isotropic properties are presented on the basis of conventionally melted alloys. This development was performed on the basis of Ti-0.44O-0.5Fe-0.08C-0.4Si-0.1Au, a near-α alloy presented by the authors for medical applications and conventional manufacturing, with yttrium and boron additions as additional growth restriction solutes. In terms of LPBF processing of CP titanium grade 1 powder, a high relative density of approximately 99.9% was obtained in the as-printed state of the volume of a small cubical sample by using optimized laser power, scanning speed, and hatch distance in combination with a rotating scanning pattern. Moreover, tensile specimens processed with these volume settings and tested in the as-printed milled state exhibited a high average yield and ultimate tensile strength of approximately 663 and 747 N/mm^2^, respectively, combined with a high average ductility of approximately 24%. X-ray diffraction results suggest anisotropic mechanical properties, which are, however, less pronounced in terms of the tested specimens. Regarding alloy development, the results show that yttrium additions lead to a considerable microstructure refinement but have to be limited due to the occurrence of a large amount of precipitations and a supposed higher propensity for the formation of long columnar prior β-grains. However, phase/texture and microstructure analyses indicate that Ti-0.44O-0.5Fe-0.08C-0.4Si-0.1Au-0.1B-0.1Y is a promising candidate to achieve lower anisotropy during LPBF processing, but further investigations on LPBF printing and Y_2_O_3_ formation are necessary.

## 1 Introduction

In medical applications, the use of titanium and titanium alloys is often desirable due to their superior mechanical properties, biocompatibility, and corrosion resistance (Geetha et al., 2009; Zhang and Chen, 2019). For example, commercially pure (CP) titanium is often used for dental implants (Froes, 2018; Gubbi and Wojtisek, 2018). Typical alloys used for biomedical applications include the (α+β)-alloys Ti-6Al-4V, Ti-6Al-4V ELI (exhibiting an extra low amount of interstitials, therefore termed “ELI”), or Ti-6Al-7Nb, all of which possess much higher strength values than CP titanium (Li et al., 2014; Zhang and Chen, 2019). Within this field, additive manufacturing, for example, via laser powder bed fusion (LPBF), became increasingly popular in the past years since medical implants or devices could be produced with much higher design freedom and, possibly, with patient-tailored geometries that could therefore improve medical treatment substantially (DebRoy et al., 2018; Davoodi et al., 2022). Consequently, there are additively manufactured implants which have already been approved for commercial exploitation or implantation (Davoodi et al., 2022). Investigations on LPBF printing of (metastable) β-alloys for implant applications, which provide good properties for this purpose especially due to their lower modulus of elasticity being more close to the human bone, are rather scarce (Zhang et al., 2023). Irrespective of the titanium alloy used, a (further) reduction in stiffness to match the properties of the human bone could also be achieved by LPBF printing of lattice structures (Chen et al., 2021; Zhang et al., 2023). However, despite the benefits that additive manufacturing has to offer, several issues have to be either considered or solved prior to the comprehensive usage of additively manufactured medical parts. Among other issues, on the one hand, unsuitable process parameters can lead to several defects in LPBF printed parts, such as, porosity due to insufficient fusing of powder particles or fusion of melt tracks with negative impact on mechanical properties (DebRoy et al., 2018), while on the other hand, anisotropic (mechanical) properties are typically expected after LPBF printing, since the unique solidifcation conditions present, the layer-wise fabrication with (partial) remelting of prior layers and the chemical composition usually lead to epitaxial growth and formation of columnar, textured prior β-grains (DebRoy et al., 2018; StJohn et al., 2018; Zhang et al., 2020a). This β-texture thereby determines the α- or α′-texture after allotropic transformation due to the Burgers orientation relationship (Barriobero-Vila et al., 2018). The resulting anisotropic properties are, however, not always desired, and many efforts have already been made to obtain (more) isotropic properties after additive manufacturing, as described later in this section in more detail.

The present study addresses both issues. On the one hand, LPBF printing of CP titanium grade 1 is investigated in detail for process-induced porosity, microstructure, and static mechanical properties. A design of experiments is used in order to establish a process window on laser power, hatch distance, and scanning speed to minimize volume porosity in the as-printed state. Such a statistical approach, which is rather seldom used in the existing literature for LPBF processing of titanium, is a better choice for the process optimization procedure in order to improve the outcome than a more non-strategic approach. Specimen characterization was performed using microstructure and phase analysis that included qualitative texture assessment as well as tensile tests and elemental analysis in terms of oxygen- and nitrogen-pickup during LPBF processing. Consequently, this study provides or summarizes a broad and extensive overview of process parameters, microstructure and defect formation, and mechanical properties. On the other hand, the first results on the development of a near-α or (α+β)-titanium alloy tailored to LPBF processing for higher strength, medical applications with lower anisotropy and higher biocompatibility than conventional biomedical (α+β)-titanium alloys are presented. In contrast to the existing or past research on anisotropy reduction during additive manufacturing as summarized below, the present study specifically focuses on developing a biocompatible alloy. The reduction in anisotropy is planned to be achieved by increasing constitutional supercooling during solidification through the use of alloying elements providing growth restriction: oxygen, carbon, iron, silicon, yttrium, and boron (Zhang et al., 2020a). In particular, the influence of different amounts of yttrium and of a small amount of boron added to Ti-0.44O-0.5Fe-0.08C-0.4Si-0.1Au is investigated by melting Ti-0.44O-0.5Fe-0.08C-0.4Si-0.1Au-(0.3/0.5/1.0)Y and Ti-0.44O-0.5Fe-0.08C-0.4Si-0.1Au-0.1B-0.1Y in order to determine a suitable alloy as the possible candidate for LPBF processing of medical implants with lower anisotropy. Ti-0.44O-0.5Fe-0.08C-0.4Si-0.1Au was chosen as the base alloy since it was already presented by the present authors as an alternative to Ti-6Al-4V or Ti-6Al-4V ELI for conventional implant or osteosynthesis product manufacturing with supposed higher biocompatibility (Haase et al., 2022) and already exhibits increased amounts of oxygen, carbon, iron, and silicon (when compared to conventional titanium alloys), which provide constitutional supercooling (Zhang et al., 2020a). Within the present study, yttrium and boron additions were made to the existing material of Ti-0.44O-0.5Fe-0.08C-0.4Si-0.1Au, whereas the amount of oxygen, carbon, iron, silicon, and gold remained unchanged except of negligible changes due to small amounts of alloying elements. The four alloys investigated were conventionally melted in laboratory scale using an electric arc furnace and studied in the as-melted state for solidification, microstructure, and hardness.

Anisotropy reduction during additive manufacturing has already been the goal of several studies. In general, (more) isotropic mechanical properties can be achieved in case equiaxed prior β-grains form during melt pool solidification and these grains are not remelted during processing of subsequent powder layers (StJohn et al., 2018; Zhang et al., 2020a). In terms of the chemical composition of an alloy, this would, among other requirements, necessitate potent solidification nuclei within the liquid and alloying elements with a high growth restriction effect, rapidly forming a constitutionally supercooled zone in front of the solidification front and activating the nuclei (StJohn et al., 2018; Zhang et al., 2020a). The alloying elements used in the present study are known as growth restriction solutes that provide constitutional supercooling in titanium (Zhang et al., 2020a) and have already been investigated in several studies on grain refinement during processes such as conventional melting and additive manufacturing. Regarding the effects of boron and yttrium, which will be studied in more detail here, studies have been performed by Nordin et al. (1987), Tamirisakandala et al. (2005), Bermingham et al. (2008), Bermingham et al. (2015), Mantri et al. (2017), Xue et al. (2019), Zhang et al. (2019), Zhang et al. (2020b), Wang et al. (2021), and Kennedy et al. (2022). Additionally, the possibility of Y_2_O_3_ particles acting as the nuclei for β-grain formation in titanium is discussed (Tian et al., 2006; Xu et al., 2020; Wang et al., 2021; Kennedy et al., 2022), among other possible influences of yttrium or Y_2_O_3_, which will be addressed later. Within the present study, the combined effects of the described alloying elements are investigated in order to develop a (more) biocompatible near-α or (α+β) titanium alloy tailored to LPBF printing. The effects of yttrium and boron additions on biocompatibility or potential toxicity were investigated by Feyerabend et al. (2010), Ebel et al. (2011), Bahl et al. (2014), Málek et al. (2014), and Weng et al. (2020). To conclude, small amounts of yttrium and boron seem to be acceptable as alloying elements.

To summarize, in regard to the described limitations and challenges, the present study provides a suitable set of processing parameters for LPBF printing of CP titanium grade 1 powder, achieving low residual porosity in the as-printed state for small samples and high static mechanical properties of strength and ductility in the as-printed milled state. Based on this, LPBF printing of small CP titanium components directly usable in applications without dynamic loads might be achievable. Moreover, the present study proposes a new titanium alloy for medical implant applications with the alloying elements oxygen, iron, carbon, silicon, gold, boron, and yttrium as the potential candidate to achieve a lower anisotropy after LPBF processing and a suspected higher biocompatibility than conventional (α+β) titanium alloys established for medical applications. Nevertheless, further investigations are necessary for LPBF processing of this alloy. In addition, the present knowledge and understanding of microstructure formation of yttrium-alloyed titanium alloys is complemented, and the current knowledge gaps and frontiers requiring further investigations are highlighted.

## 2 Materials and methods

### 2.1 LPBF

#### 2.1.1 CP titanium powder

Spherical CP titanium powder of two production batches was obtained from ECKART TLS GmbH, Germany, with a chemical composition of Ti-0.174O-0.016C-0.12Fe and Ti-0.15O-0.012C-0.19Fe and a D50 value of approximately 31 and 27 µm and D90 value of approximately 48 and 42 μm, respectively, according to the provided inspection certificate 3.1 (EN 10204). Consequently, both chemical compositions correspond to CP titanium grade 1 according to ASTM F67 (0.08% carbon, 0.20% iron, and 0.18% oxygen maximum) (ASTM International, 2013b) in the as-received state. Powder sieving was performed under a protective argon atmosphere using meshes with mesh widths of 45 µm and 63 μm.

#### 2.1.2 Process parameter optimization

For volume process parameter optimization, a Doehlert design (Doehlert, 1970) was used analogous to the studies of Perevoshchikova et al. (2017), who used this experimental design successfully for LPBF printing of a nickel-based superalloy. Within the present study, the effects of the laser power, scanning speed, and hatch distance (distance between parallel scan tracks) on the porosity was studied using the first alloy batch (reused, sieved powder). These process parameters varied between 40 and 360 W (seven variations in terms of the Doehlert matrix used), 100 and 2,000 mm/s (five variations), and 50 and 130 µm (three variations), respectively. The parameter ranges were chosen with respect to process parameters described in the literature for LPBF printing of CP titanium and to a process parameter set provided by the machine manufacturer. In order to provide a comprehensive analysis, all three parameter ranges were chosen to be rather large to cover a wide variety of process parameters. Since it was assumed that the laser power has the most significant influence on the porosity, seven variations were used for this parameter within the Doehlert design, whereas five and three (assumed to have the least influence) were applied for the scanning speed and hatch distance, respectively. Cubical samples with an edge length of approximately 10 mm (excluding printed support structures) were printed using a SLM^®^125 LPBF machine from SLM Solutions Group AG, Germany. A non-rotating stripe pattern with stripes perpendicular to the gas flow direction, a titanium substrate plate heated to 200°C, an argon gas flow velocity of 6 m/s (flow velocity measured within the piping of the LPBF machine), and a layer thickness of 30 µm were used. Regarding the border process parameters, which can be set independently, the laser power and scanning speed also varied according to the Doehlert design in conjunction with the volume process parameters. Two borders were printed after performing the hatching of the volume. These border process parameters are not of relevance in volume process parameter optimization, since the border was excluded during porosity measurements (see Section 2.4) but were studied in order to obtain a suitable combination. Other parameters affecting border and volume processing, but which were not seen to be relevant for the present study, were either kept according to the values provided by the LPBF machine manufacturer or set to values that were assumed to be appropriate for the experiment. Before the LPBF process started, the building chamber was flushed several times with argon of purity 99.999%. The process was started with an oxygen value of approximately 0.04%, measured by using the machine software. Due to the comparable small amount of powder used for printing, the surplus powder of the overflow container was reused once without prior sieving during the printing of the specimens. The hold time until continuation of the print job is negligible as described later. The resulting porosity was optically determined on metallographic samples as described in Section 2.4.

In order to calculate the response surface, the procedure of Perevoshchikova et al. (2017) was applied. A second order polynomial function was used to describe the influence of the three process parameters on the porosity, *P*:

$$P=aX_{1}+bX_{2}+cX_{3}+dX_{1}^{2}+eX_{2}^{2}+fX_{3}^{2}+gX_{1}X_{2}+hX_{1}X_{3}+iX_{2}X_{3}+j,$$ (1)

with *X*_1_, *X*_2_, and *X*_3_ being the coded values of the scanning speed, laser power, and hatch distance, respectively (for details on Doehlert design, see Doehlert, 1970). The coefficients *a* till *j* of Eq. 1 were determined by a multiple linear regression analysis using an ordinary least squares approach. Subsequently, the stationary point of this response surface and its type were calculated, as described by García Campaña et al. (1997) and Ferreira et al. (2004), by solving the corresponding equation system and using the Lagrange criterion, respectively. The obtained coded values of the laser power, scanning speed, and hatch distance were then transformed to their experimental values. These were regarded to be the result of the optimization procedure (optimized process parameters). For visualization, Eq. 1 was transformed in the experimental domain. All mathematical operations and visualizations were performed using the MATLAB software (The MathWorks Inc, 2022a) and the Statistics and Machine Learning Toolbox (The MathWorks Inc, 2022c) with a self-written script and existing functions.

### 2.2 Verification, microstructure analysis, and tensile specimens

After process parameter optimization, two additional print jobs were performed with the second powder batch, sieved prior to the first use, in order to verify the obtained optimized process parameters and to perform microstructural analyses, phase analysis with qualitative texture assessment, and tensile tests. Two print jobs were necessary since unsuitable border process parameters were used during the first printing (border laser power and scanning speed were set to the optimized volume laser power and scanning speed, respectively), leading to defects such that the printing of several samples had to be canceled. Among the other samples used for experiments not covered by the present study, the cubical samples with the same geometry as for process parameter optimization and tensile specimen blanks were printed. Additionally, in each print job, two small vertical rectangular rods of approximate dimensions 3.65 × 3.65 × 8 mm^3^ (excluding support structures with a height of 4 mm and specimen numbering) were printed on opposite ends of the substrate plate in the gas flow direction. A small disk with a height of approximately 1.1 mm was cut of each sample directly above the support structures, resulting in specimens with a weight of approximately 59–63 mg. These specimens were used for chemical analysis in order to determine the oxygen, nitrogen, and hydrogen contents of LPBF printed parts. These measurements were conducted at the Institute of Metallic Biomaterials, Helmholtz-Zentrum Hereon, Germany, using a LECO^®^ ONH836 elemental analyzer. The average values and empirical standard deviations were used to evaluate the obtained results. Regarding both print jobs and all specimens, the calculated laser power (213.2 W), scanning speed (1,427 mm/s), and hatch distance (0.058 mm) according to the process parameter optimization were used for volume processing. Additionally, however, a rotating scanning pattern was applied with a limitation window of 90°, an angle start value of 0°, and an angle increment of 67°. The substrate plate temperature and gas flow velocity were unchanged. For the second print job, the border laser power and scanning speed were adjusted to 93.32 W and 1,050 mm/s, respectively, on the basis of the print job performed for process parameter optimization.

**FIGURE 1:**
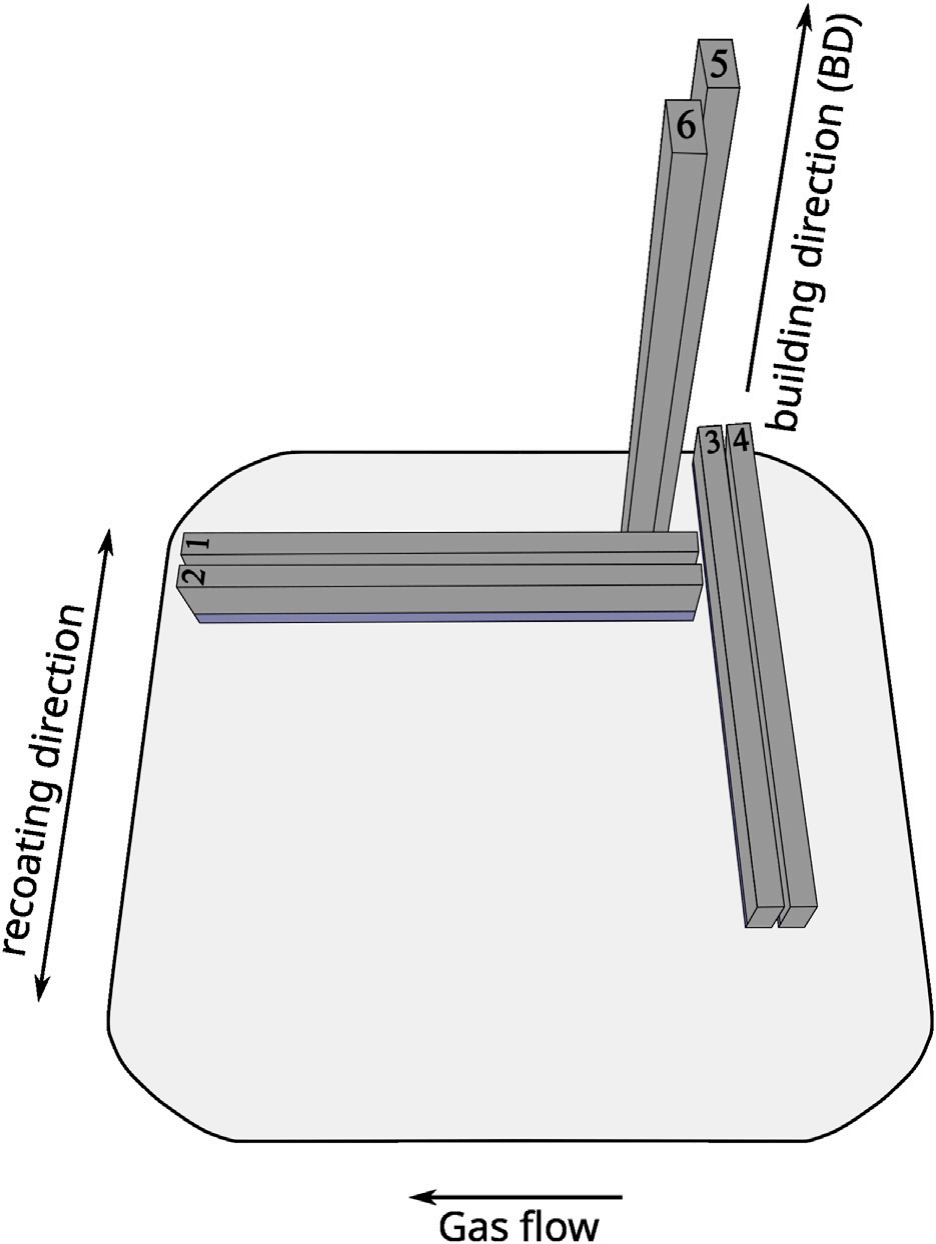
Schematic drawing of the orientation and approximate positioning of tensile specimen blanks on the substrate plate for gas flow, recoating, and building direction (additional specimens not shown).

Verification of the optimized process parameters was performed by analyzing one of the printed cubical samples of the first print job using the same approach as for process parameter optimization. The same specimen was subsequently used for microstructure analysis. For comparison, the microstructure of a similar cubical sample of the second print job was investigated as well, which was also used for phase analysis by X-ray diffraction. For tensile tests, flat tensile specimen blanks were printed, which were pairwise differently oriented for gas flow and printing direction as illustrated in Figure 1 in order to study a possible orientation dependence of the mechanical properties. Since printing of all specimen blanks of the first print job had to be canceled due to unsuitable border process parameters, only the specimens of the second print job were used for the tensile tests. During processing, the surplus powder of the overflow containers had to be sieved and reused due to the high length of the verticaly printed specimens. Consequently, the print job was halted thrice. One of these holds lasted comparatively longer for approximately 4 h 45 min than the others that lasted less than 10 min. A possible influence of this long hold or processing time on the microstructure due to substrate plate heating at 200°C will be analyzed in the Results section.

### 2.3 Alloy melting

Four different alloys were melted conventionally and subsequently studied, Ti-0.44O-0.5Fe-0.08C-0.4Si-0.1Au-(0.3/0.5/1.0)Y and Ti-0.44O-0.5Fe-0.08C-0.4Si-0.1Au-0.1B-0.1Y, using pre-cast Ti-0.44O-0.5Fe-0.08C-0.4Si-0.1Au material (Haase et al., 2022) and pieces of elemental yttrium and boron of purity 99.9% and 99.5%, respectively. Melting was performed with a small laboratory size electric arc furnace, type MAM (Edmund Bühler GmbH), using argon of purity 99.999% and a melting pressure of approximately 700 mbar, leading to small button-shaped specimens with similar weight of approximately 4 g. Prior to melting, the chamber was evacuated four times to a pressure below 1 ⋅ 10^–2^ mbar and flushed with argon in between. Each specimen was turned and remelted thrice to achieve a homogeneous chemical composition. Except for Ti-0.44O-0.5Fe-0.08C-0.4Si-0.1Au-0.1Y, the electric arc current was partially increased in some melting steps, since solidification started rapidly, once the arc was moved. Additionally, during melting of Ti-0.44O-0.5Fe-0.08C-0.4Si-0.1Au-0.1B-0.1Y, the chamber had to be evacuated again prior to the second and third remelting due to overheating of the furnace. Prior to each melting, a small sample of titanium foil was melted first to possibly trap oxygen that remained within the chamber. In order to establish comparable solidification conditions, sample cooling was kept the same, by keeping the specimens on the water-cooled copper crucible plate for approximately 4 min in total, as the water cooling kept on the first 3 min. A sample of Ti-0.44O-0.5Fe-0.08C-0.4Si-0.1Au, processed the same way as already described and discussed by Haase et al. (2023), will be used as the reference specimen.

### 2.4 Metallographic specimen preparation and microstructure analysis

All LPBF-printed samples were first cut of the substrate plate using a manually operated abrasive cutter with water cooling. Specimens were then sectioned using a precision cutting machine with the plane of sectioning being parallel to the building and gas flow direction and at the approximate middle of the specimen. Melted specimens of the boron- or yttrium-containing alloys were cut parallel to the expected macroscopic solidification direction in the as-cast state. The CP titanium rod material, used for comparison during the microstructure analysis and tensile tests (see Section 2.6), was studied for its cross section. All specimens were subsequently warm embedded in EpoMet™ G (Buehler) and BAKELIT (ATM Qness) mounting compound (the latter used as filler material) and then ground, polished, and etched according to standard metallographic specimen procedures. Etching was performed for approximately 10 s using a mixture of 86 vol% H_2_O, 4.5 vol% HNO_3_, 12 vol% H_2_O_2_, and 5 vol% HF. Regarding porosity measurements, grinding was performed by using SiC grinding papers of sizes P180, P240, P320, P400, P600, P800, P1200, and P2500 on an automatic grinding machine (ATM SAPHIR 550). The P1200 and P2500 grinding papers were thereby pretreated with wax. Polishing was conducted using MasterPrep™ (Buehler) polishing suspension with a particle size of 0.05 µm together with oxalic acid for approximately 10 min followed by 1 min rinsing with tap water.

The porosity of the samples was determined via optical analysis on polished and cleaned (soap water and ethanol) samples. A reflected light microscope (ZEISS Axio Imager.M2m) was used in the bright field mode for image capturing, and the Fiji software (Schindelin et al., 2012) was used for porosity measurements. For each specimen, nine images were obtained with ×100 magnification in a pre-defined 3 × 3 grid, excluding borders and support structures. During the image analysis, the upper threshold value was individually set for each image and determined by sight. This and some residual ethanol spots or artifacts from the metallographic specimen preparation left on the specimens' surface lead to a slight uncertainty or error on the measured porosity. This uncertainty is hardly quantifiable, since the error in measurement due to choice of the upper threshold value will be a function of the individual porosity within the analyzed picture and the pores' contour sharpness, which is the outcome of the specimen preparation procedure. This contour sharpness and the amount of residual polishing artifacts can be specimen dependent. Regarding the residual ethanol spots, the relative error could possibly be comparably high if the total porosity is low. Accordingly, the measured porosities or relative densities should be seen as approximate values. Nevertheless, two decimals are used for measured porosities, since otherwise fully dense specimens would be partly indicated by the results.

For microstructural investigations, a reflected light microscope (Olympus BX51M) was used in order to obtain panorama images of the samples, which consist of several individual images taken with ×50 or ×100 magnification, which have been captured and stitched together automatically using the Stream Motion software (Olympus Corporation, 2011). In addition, a tabletop SEM microscope (Hitachi TM3000) operated with an acceleration voltage of 15 kV (with and without increased beam current) and equipped with a BSE detector and an EDS system (QUANTAX 70, Bruker Nano) was used to obtain high-magnification images and perform chemical analyses.

### 2.5 Phase analysis

The phase analysis was performed via X-ray diffraction (XRD) with a GE XRD 3003 PTS diffractometer in the Bragg–Brentano setup using an X-ray tube with copper anode operated with an electric voltage and current of 40 kV and 40 mA, respectively, and a nickel filter. Ti-0.44O-0.5Fe-0.08C-0.4Si-0.1Au (Haase et al., 2023), Ti-0.44O-0.5Fe-0.08C-0.4Si-0.1Au-0.1B-0.1Y, and Ti-0.44O-0.5Fe-0.08C-0.4Si-0.1Au-1.0Y in the as-cast state and LPBF-printed CP titanium grade 1 in the as-printed condition were analyzed as embedded, etched, or polished samples. Regarding Ti-0.44O-0.5Fe-0.08C-0.4Si-0.1Au-1.0Y, the point focus of the X-ray tube was used with a 1 mm pinhole collimator, 6 mm anti scatter, and 2 mm detector slit. No secondary Soller slit was used. The measurement was performed with Bragg angles of 28°–80°, a step width of 0.01°, and a measuring time of 5 s. To be more precise, the beam path consisted of the following slits, viewed from the X-ray tube: Primary side: (1) 3 mm slit, (2) soller slit, (3) 2 mm slit. Secondary side: (4) 2 mm anti scatter slit, (5) soller slit, (6) 0.5mm detector slit. These measurements were performed for Bragg angles of 34°–80°, a step width of 0.005°, and an integration time of 4 s. All as-cast specimens were identically oriented during the measurement.

The analysis was performed using the OriginPro software (OriginLab Corporation, 2023) for background correction and visualization and CMPR software (Toby, 2005) with the PDF-2 database, release 2005, for peak identification. For this, the PDF-2 database entries 441294 (α-Ti), 441288 (β-Ti), 50700 (TiB), 411105 (Y_2_O_3_), and 331458 (hexagonal pure yttrium) were used. The α-Ti database entry was also used for a qualitative texture assessment by comparing measured and ideal intensity ratios.

### 2.6 Mechanical tests

Standard flat tensile specimens according to DIN EN ISO 6892-1:2017-02 (DIN Deutsches Institut für Normung e.V, 2017) were manufactured out of the printed tensile specimen blanks. Since no subsequent heat treatment was performed, all specimens exhibited residual stresses, which led to bending during milling. Consequently, the specimens had to be deformed plastically during manufacturing, which might have an effect on the measured mechanical properties due to work hardening. For comparison, four round tensile specimens of type B5 × 25 according to DIN 50125:2016-12 (DIN Deutsches Institut für Normung e.V, 2016) were manufactured out of conventional CP titanium rod material with chemical composition Ti-0.14O-0.01C-0.09Fe. Tensile tests were performed at room temperature according to DIN EN ISO 6892-1:2017-02 (DIN Deutsches Institut für Normung e.V, 2017), method B, using a stress speed of 10 MPa/s within the elastic regime, a test speed of 0.006 1/s, and an initial stress of 5 MPa. A hysteresis loop was used for a better calculation of the modulus of elasticity and consequently the yield strength. The latter was defined as the strength at a plastic elongation of 0.2%. Mechanical tests and the calculation of mechanical properties were performed using the testXpert^®^ II (Zwick GmbH and Co, 2007) (rod material) or testXpert^®^ III (ZwickRoell GmbH & Co. KG, n.d.) software. The average values and empirical standard deviations were used for evaluation.

Regarding the yttrium- and boron-containing alloys, the Vickers hardness tests were performed on the embedded, etched, or polished samples to investigate the mechanical properties in the as-cast state. These tests were conducted using a LECO^®^ AMH43 automatic hardness testing system with the LECO^®^ LV100AT Vickers hardness tester and AMH43 software (LECO Corporation, 2018). Five indentations using a test force corresponding to HV 10 and a holding time of 15 s were evaluated for each specimen. These indentations were placed vertically in a straight line along the sample's entire height, roughly corresponding to the macroscopic solidification direction (see the Results section). A magnification of ×20 was used to survey each indent. The average values as calculated by the software and empirical standard deviations were used for evaluation. The typical accuracy obtained during the Vickers hardness tests was approximately 3%. In terms of Ti-0.44O-0.5Fe-0.08C-0.4Si-0.1Au-(0.3/0.5/1.0)Y, the minimum distance between individual indents as required for light metals according to DIN EN ISO 6507-1:2018-07 (DIN Deutsches Institut für Normung e.V, 2018) was not satisfied. This was especially the case for Ti-0.44O-0.5Fe-0.08C-0.4Si-0.1Au-1.0Y. However, a second hardness test with horizontally aligned and sufficiently distanced indents for this specimen led to similar average hardness within the empirical standard deviation or typical accuracy of the method. Significant influences are therefore not expected.

## 3 Results

### 3.1 LPBF of CP titanium

#### 3.1.1 Process parameter optimization

**FIGURE 2:**
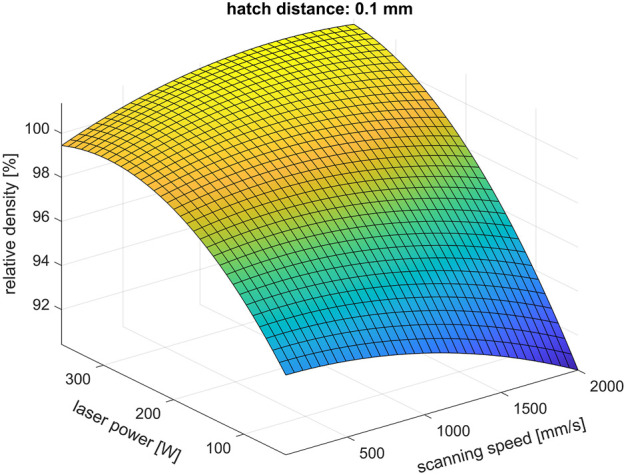
Relative density depending on scanning speed and laser power for a hatch distance of 0.1 mm during LPBF printing of CP titanium grade 1 (calculated response surface). Only 12 of the 13 specimens printed within the Doehlert design were used for regression analysis.

The 13 specimens printed as part of the Doehlert design exhibit an average porosity between approximately 0.01% and 47%. The regression analysis performed with all these 13 results led to a comparable low *R* ^2^ value of approximately 0.79 with high differences between the calculated and actual porosities (residuals) of up to approximately 10% in terms of the absolute values. The highest residual is particularly present for the specimen exhibiting the highest measured porosity of approximately 47%, which was LPBF processed with the lowest energy density of approximately 10 J/mm^3^. In order to achieve better approximation for specimens with more realistic process parameters, this specimen was omitted during further calculation, resulting in a much better regression result for the remaining 12 specimens with an *R* ^2^ value of approximately 0.93, an adjusted *R* ^2^ value of approximately 0.59, and residuals lower than 1.1% in terms of the absolute values. The corresponding response surface is depicted in Figure 2, showing the relative density as a function of the scanning speed and laser power for a fixed hatch distance of 0.1 mm. The *p*-values of the calculated coefficients of Eq. 1, which indicate whether the respective term has a significant influence on the response (The MathWorks Inc, 2022b), are between approximately 0.1 and almost 1. The laser power, hatch distance, or scanning speed therefore does not have a significant influence on porosity when a significance level lower than 10% is used. Nevertheless, on the basis of Figure 2, it can still be assumed that the laser power is an important parameter, since the relative density seems to vary noticeably with changes in the laser power. A porosity of several percent would, thereby, not be acceptable. Regarding the specimen, which was omitted during regression analysis, the response surface predicts a porosity of only approximately 6%. The established response surface cannot therefore describe the porosity during LPBF printing completely. The stationary point of the established equation is a saddle point with a calculated relative density of approximately 101% and a scanning speed, laser power, and hatch distance of approximately 1,427 mm/s, 213.2 W, and 0.058 mm, respectively, corresponding to an energy density of approximately 86 J/mm^3^. This parameter set is the result of the volume process parameter optimization conducted on CP titanium grade 1 powder for small cubical samples and a non-rotating scanning pattern.

Regarding border processing, numerous combinations of laser power and scanning speed led to coating errors detected by the LPBF machine software. Two parameter sets led to considerable surface defects, damaging the recoater lip (polymer) and affecting subsequent powder layers. Three specimens showed little or few coating errors.

During subsequent verification of the established set of optimized volume process parameters using, additionally, a rotating scanning pattern (see Section 2.2, which can lead, among other possible influences on properties or microstructure to lower porosity (Thijs et al., 2010)), an average porosity of approximately 0.02% was measured. The highest measured porosity (single image) was approximately 0.09%. It is expected that the actual porosity is slightly higher than the average value, since additional microstructural images show larger porosity, which were not considered during porosity measurements. Process parameter optimization was thus successful, at least for small cubical samples. However, flat printed plates partially showed a very low surface quality, which seemed to be dependent on the specimens’ position on the substrate plate. Consequently, the obtained optimized volume process parameters seem to be valid for small specimens only. A different, conical sample processed with the same settings and studied tentatively via computed tomography showed five recognizable pores within the sample's volume with a maximum size of roughly 30 µm. A precise size determination was not possible. Few other pores were located close to the specimen’s surface with one pore exhibiting a comparable size of roughly 100 µm. This porosity is believed to be caused by border processing. In general, the optimized volume laser power and scanning speed were (in combination with additional parameters affecting border processing) not sufficient for border processing since several specimens that included two additional cubical samples with the same geometry exhibited tips on the border, damaging the recoater lip and negatively influencing subsequent powder layers. This is especially the case for long edges that are parallel to the gas flow direction and also seemed to be position dependent.

Apart from this combination of volume process parameters, a processing window for LPBF printing of CP titanium grade 1 is established according to Figure 2, suggesting that several combinations of laser power, scanning speed, and hatch distance can lead to a high relative density in the as-printed state. This is supported by specimens of the Doehlert design; for example, despite the great difference in the energy density of 71 J/mm^3^ (laser power, scanning speed, and hatch distance of 200 W, 1,050 mm/s, and 90 μm, respectively) when compared to 195 J/mm^3^ (laser power, scanning speed, and hatch distance of approximately 307 W, 1,050 mm/s, and 50 μm, respectively), both specimens exhibit very low porosity of only approximately 0.01% and 0.02% with an empirical standard deviation of approximately 0.01% each.

#### 3.1.2 Microstructure, phase, and elemental analyses

**FIGURE 3:**
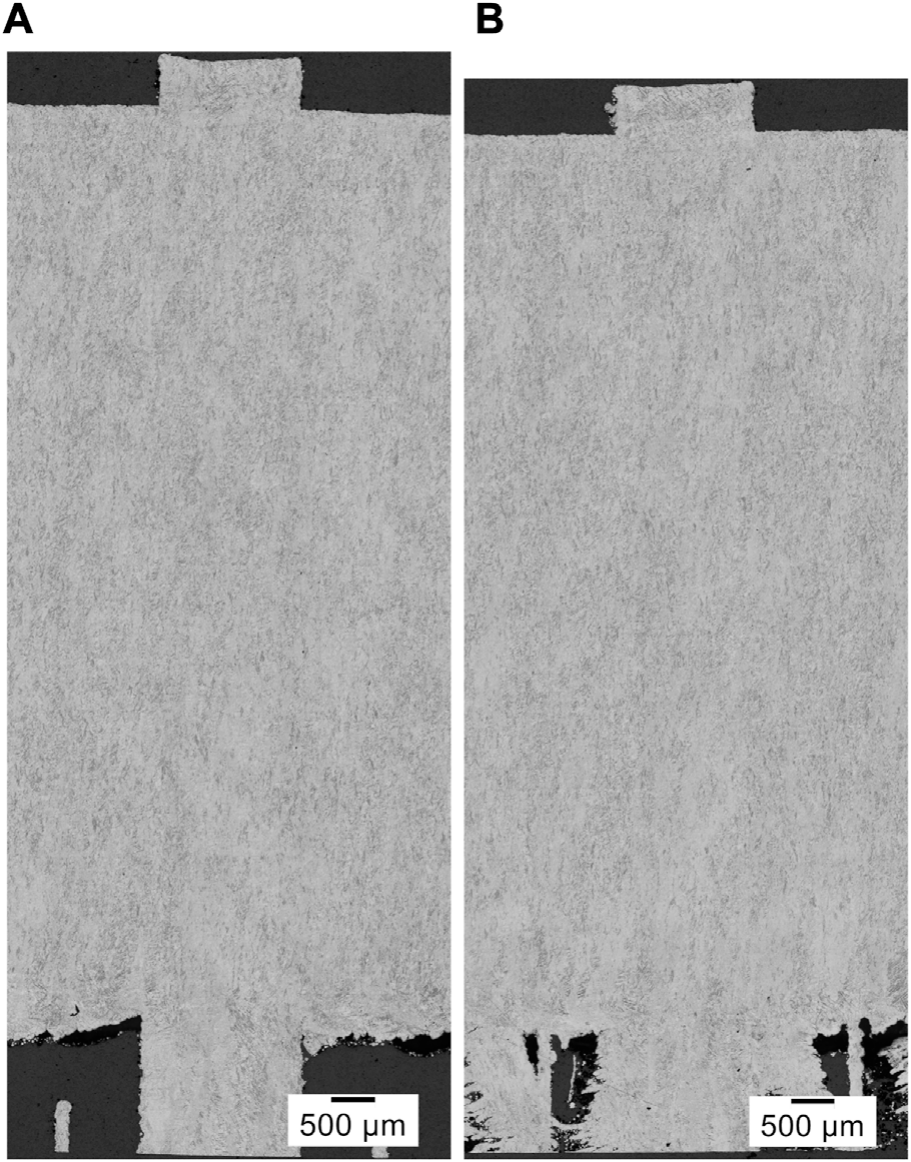
Cropped panorama images of the microstructure of LPBF printed CP titanium grade 1 in the as-printed state, printed with optimized volume process parameters but different border parameters. **(A)** Specimen of the first additional print job. **(B)** Specimen of the second additional print job.

Figure 3 shows cropped panorama images of LPBF-processed CP titanium grade 1 in the as-printed state of a specimen of the first (Figure 3A) and second (Figure 3B) print jobs. As can be seen, a substantial columnar microstructure cannot be observed. Prior β-grain boundaries are not visible, but regions with similar orientations of α- or α′-grains do partially exhibit a columnar appearance, suggesting the presence of elongated, long prior β-grains aligned with the building direction, as expected. According to these panorama images, the microstructure appears to be homogeneous throughout the samples that include the last scan tracks at the top of each specimen. The latter suggests that the microstructural changes due to platform heating did not occur *in situ* during LPBF printing. Moreover, it can be seen that significant differences between both analyzed specimens are not present, which is supported by SEM analyses, although the second print job lasted much longer (more than 43 h) than the first (more than 9 h). Consequently, it is expected that the tensile samples printed during the second print job exhibit a similar microstructure.

**FIGURE 4:**
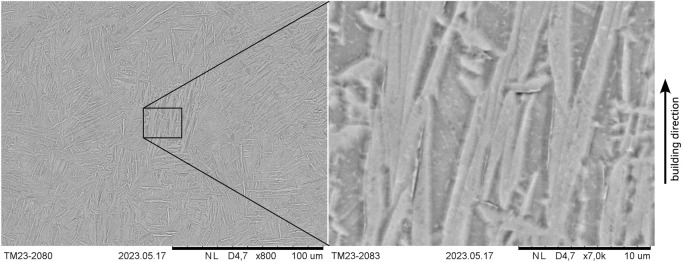
Microstructure (SEM and BSE detector) of LPBF-processed CP titanium grade 1 in the as-printed state (specimen printed together with tensile specimens).

Figure 4 shows the SEM images (BSE detector) of the as-printed microstructure at the approximate center of the specimen that is being printed together with the tensile specimens. At lower magnifications and in accordance with the optical microscope images, a needle-like martensitic microstructure seems to be present but appears to be heterogeneous in size and shape and seems to contain broad structures as well. Regarding the latter, further features or grain boundaries within these structures are not visible at lower magnifications. However, as can be seen in the high-magnification image of Figure 4, broader structures, at least partially, consist of almost parallel, finer plates or needles. The smaller substructures are, nevertheless, not always recognizable, which is also evident from Figure 4. By contrast, much smaller, round, white particles are supposed to be artifacts from metallographic specimen preparation. In addition, pitting corrosion seems to have taken place at the grain boundaries due to etching, which is often observed with this etchant, further indicating the presence of smaller structures being not visible with lower magnification.

In general, due to the very high cooling rates during LPBF printing (DebRoy et al., 2018), a martensitic β to α transformation would be expected during cooling. Although *in situ* decomposition of martensite might be possible (Xu et al., 2015), microstructural differences between both specimens and along the the building direction that included the last laser tracks were not observed, as has already been described, suggesting that no martensite decomposition took place. Moreover, the microstructure exhibits similarities with massive martensite, which is characterized by approximate parallel α-plates forming larger packets and microstructural areas, in which the grain boundaries are hardly visible (Lütjering and Williams, 2007). This type of martensite is thereby present, among others, in pure titanium and alloys with a very low amount of alloying elements (Lütjering and Williams, 2007). Therefore, it is concluded that the LPBF-processed samples consist of massive martensite. In the literature, the microstructure observed after LPBF printing of CP titanium has also been described as plate-like or lenticular α′-laths (Wysocki et al., 2017), α-grains, shaped as plates, or acicular α′ (depending on process parameters) (Attar et al., 2014) and α′-laths or acicular α′ (Attar et al., 2017). It is also reported that different α′-structures can be present within a sample (Attar et al., 2017).

**FIGURE 5:**
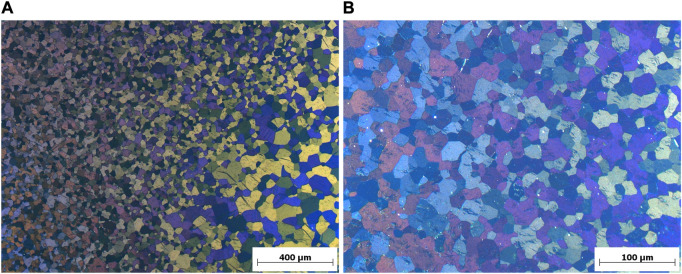
Polarized light images of the microstructure (cross section) of conventional rod material of CP titanium grade 1. **(A)** Overview image showing coarse and finer equiaxed grains. **(B)** Higher magnification image of the approximate center of the rod’s cross section showing fine equiaxed grains.

By contrast, the conventional rod material used as comparison during tensile testing exhibits equiaxed α-grains, as can be seen in Figure 5, which depicts an overview and a higher magnification, polarized light image of the microstructure. Consequently, the material was recrystallized after deformation. Figure 5A shows that the grain size varies noticeably. In this case, a rather extreme case with very coarse equiaxed grains in the bottom right corner, corresponding to the edge of the rod's cross section, and much finer grains on the left hand side are shown. By contrast, the higher magnification image in Figure 5B shows fine equiaxed grains in the approximate center of the rod. When compared to the microstructure of the LPBF-processed sample (Figure 4) it is evident that a much finer but different microstructure is obtained during LPBF processing, as expected.

**FIGURE 6:**
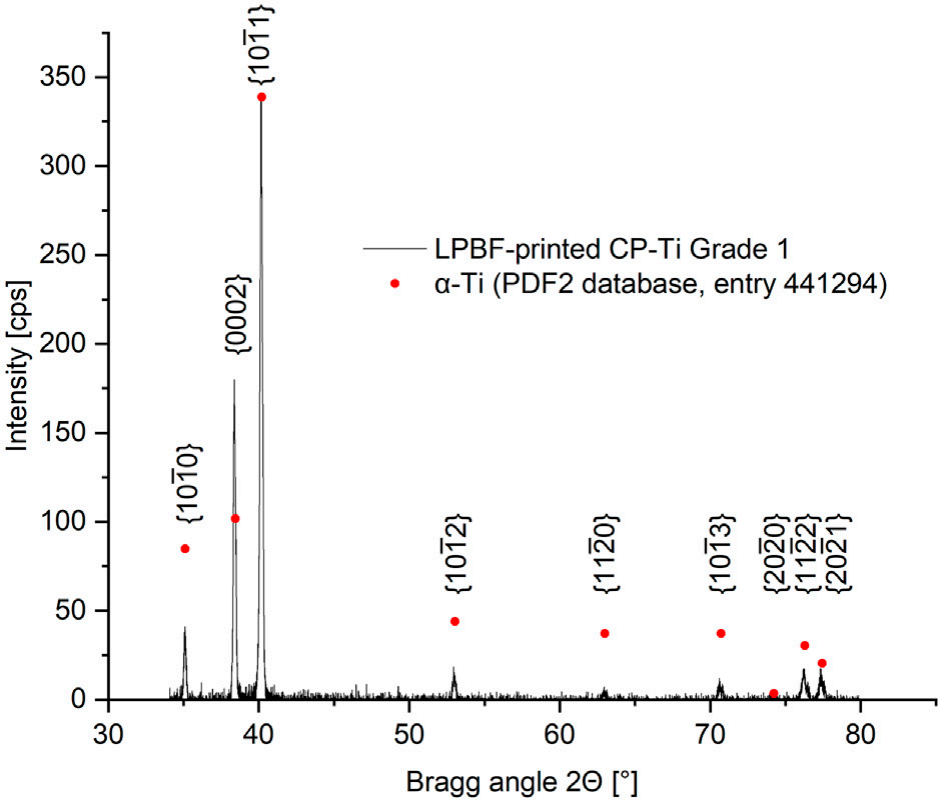
Diffractogram of CP titanium grade 1, LPBF processed with optimized volume process parameters, with assigned lattice planes. Peak positions and rescaled intensities for pure α-titanium, obtained from the PDF2 database are indicated.

Figure 6 shows the diffractogram measured for LPBF-printed CP titanium grade 1, processed with the established optimized process parameters. Additionally, the peak positions and intensities obtained for α-titanium from the PDF2 database are shown. The intensity of the main α-peak $\left\{10\bar{1}1\right\}$ was thereby set to the maximum value measured, and the intensities of the other peaks were rescaled accordingly. These values or their ratios, therefore, represent an ideal, texture free sample. As can be seen, remarkable differences exist between the measured and ideal peak intensities or ratios. For example, the intensity obtained for the {0 0 0 2} basal planes is increased. Consequently, more basal planes seem to be parallel to the specimen's surface and thus parallel to the building and gas flow direction, indicating a texturized sample.

It is important to note that no peak separation in individual reflections of the Cu-K_α1_ and K_α2_ radiation was detectable, since the rather large receiving slit used (necessary for obtaining enough intensity for higher indexed planes) resulted in broader peaks. The measured peak intensities are, therefore, a combination of the K_α1_ and K_α2_ peaks. Regarding the texture-free sample, this could affect the intensity ratios obtained in dependence on the size of the receiving slit, since the visible K_α1_ and K_α2_ peaks separation can occur at higher Bragg angles depending on the receiving slit's size (Spieß et al., 2019). The exact peak overlap and consequently the combined peak intensity and intensity ratios depend on the Bragg angle (which is a result of the Bragg equation). In this case, it is reasonable to suggest that this effect can be neglected for low Bragg angles.

Regarding the elemental analysis, the measured average oxygen, nitrogen, and hydrogen contents and empirical standard deviations are listed in Table 1 for the first and second print jobs. When compared to the initial oxygen, nitrogen, and hydrogen contents of the powder of 0.15%, 0.010%, and 0.001%, respectively, according to the provided inspection certificate 3.1 (EN 10204), all values seem to have increased substantially. The measured oxygen contents are beyond the limits of CP titanium grade 1 (0.18 wt%) and close to the limits of CP titanium grade 2 (0.25%) as specified by ASTM F67 (ASTM International, 2013b). Especially the nitrogen contents are very high, exceeding the requirements of any titanium alloy specified by ASTM B348 (ASTM International, 2013a). Since only two specimens were tested for each print job, the results might not be fully representative. Although, in principle, the oxygen, nitrogen, and hydrogen uptake is expected to decrease with the build height, since the process atmosphere should get purified during the print job. Decreasing oxygen values of the processing atmosphere are always seen at the beginning of the print jobs as measured by the build-in oxygen sensors of the LPBF machine. It is therefore expected that the results presented in Table 1 can be considered as approximate maximum values achieved in specimens of these two print jobs, since the analyzed samples corresponded to material directly above the support structures. An increase in oxygen and nitrogen contents during LPBF processing of CP titanium was also determined by Na et al. (2018).

**TABLE 1:** Results of elemental analysis (average values) with standard deviations in brackets.

	Oxygen [µg/g]	Nitrogen [µg/g]	Hydrogen [µg/g]
First print job	2,223 (153)	544 (33)	63 (3)
Second print job	2,394 (33)	668 (18)	70 (5)

#### 3.1.3 Mechanical properties

**FIGURE 7:**
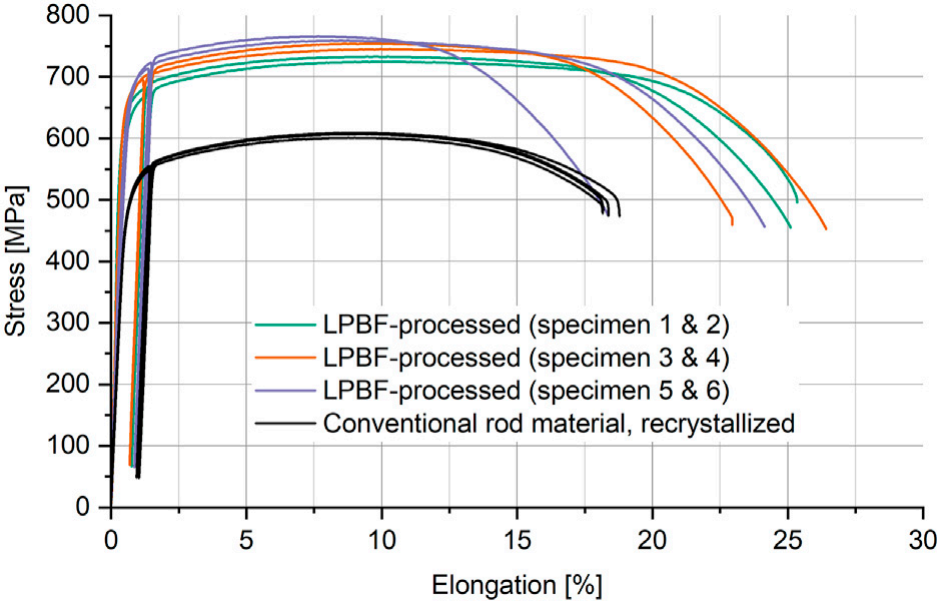
Tensile test results of LPBF printed CP titanium grade 1 (optimized volume process parameters) and of conventional recrystallized CP titanium grade 1 rod material.

Figure 7 shows the obtained stress–strain curves of LPBF-printed (optimized process parameters) and of conventional, recrystallized rod material of CP titanium grade 1. The visible drop in stress and elongation in each depicted stress–strain curve is due to the use of a hysteresis loop during testing, as described in the Methods section. Table 2 lists the obtained results in terms of the average Young's modulus, yield strength (YTS), ultimate tensile strength (UTS), and elongation after fracture (A). Standard deviations are given in brackets. Regarding LPBF printing, the results are arranged according to the three different specimen directions as indicated in Figure 1. By averaging all six specimens, LPBF-printed CP titanium grade 1 exhibits YTS and UTS of approximately 663 and 747 N/mm^2^ with empirical standard deviations of approximately 16.8 and 15.8 N/mm^2^, respectively. This is much higher than the YTS and UTS of the tested conventional recrystallized rod material of 494 (2.4) and 606 (3.6) N/mm^2^, respectively. Ductility seems to be increased as well, but since all the round tensile specimens of the rod material fractured at the end of the measuring section, their average elongation after fracture of approximately 17.9 (0.3)% was possibly underestimated. Analogically, specimen 5 of the LPBF-processed flat samples fractured at the very end of the measuring section, consequently leading to a much shorter curve within the plastic regime in Figure 7. Omitting this specimen, LPBF-processed CP titanium grade 1 exhibits an elongation after fracture of approximately 24.4%, with an empirical standard deviation of 1.3%.

**TABLE 2:** Tensile test results (average values) with standard deviations in brackets for LPBF-processed and conventional recrystallized rod material of CP titanium grade 1.

	Specimen	E	YTS	UTS	A
		[GPa]	[N/mm^2^]	[N/mm^2^]	[%]
LPBF, as print	1 and 2	121 (3.5)	643 (13.5)	729 (5.9)	24.9 (0.1)
	3 and 4	113 (5.2)	672 (1.4)	750 (6.5)	24.3 (2.5)
	5 and 6	115 (5.7)	674 (1.6)	762 (4.9)	23.8 (—)^a^
Rod material, recrystallized	1–4	102 (0.8)	494 (2.4)	606 (3.6)	17.9 (0.3)^b^

^a^ Value of the representative specimen only.

^b^ Possibly underestimated due to fracture location.

In general, based on the described standard deviations, the conventional rod material seems to exhibit more even mechanical properties than the LPBF-processed samples. This is, however, not necessarily the case if individual LPBF specimen directions are considered, as can be seen in Table 2. Excluding the modulus of elasticity, only the yield strengths measured for specimens 1 and 2 show remarkable differences. Moreover, as can be seen in Table 2 and Figure 7, different specimen directions seem to exhibit different mechanical properties. The amount of specimens tested for each specimen direction is, however, not sufficient to allow a reliable statement. This is further discussed in Section 4.1.

### 3.2 Alloy development

#### 3.2.1 Microstructure and hardness analysis

Regarding the furnace used in the present study, solidification of alloys usually starts at the bottom of specimens near the water-cooled copper crucible plate, where a small layer of unmolten material exists during melting. However, for the alloy with 0.5Y, an additional solidification front was partly visible, originating from the specimens' top. Moreover, particles were partly present within the melt, which—if at all—dissolved only slowly. Both were especially the case for the alloy with 1.0Y, with particles and two solidification fronts being present during all remelting steps, the latter moving toward each other. For the alloy containing 0.5Y, after last remelting, solidification started additionally at such an unmolten particle with the solidification front extending in all directions. Both were not the case in terms of Ti-0.44O-0.5Fe-0.08C-0.4Si-0.1Au-0.1B-0.1Y. Solidification of the 0.3Y alloy was not studied thoroughly, so precise statements on this alloy are not possible. Nevertheless, after the last remelting step, solidification seemed to have ended at the specimen's tip.

It has to be noted that during the SEM analysis (BSE detector) of the specimen's bottom of Ti-0.44O-0.5Fe-0.08C-0.4Si-0.1Au-0.1B-0.1Y, brighter areas were present with the EDS analysis showing increased Y content. Therefore, it has to be assumed that the added yttrium was not melted completed, thus leading to a lower yttrium content than 0.1%. A global EDS analysis of this specimen in order to determine its yttrium content was not possible due to the low amount of the alloying element possibly being lower than the detection limit during the EDS analysis, since a Y-peak was not visible in the spectrum.

**FIGURE 8:**
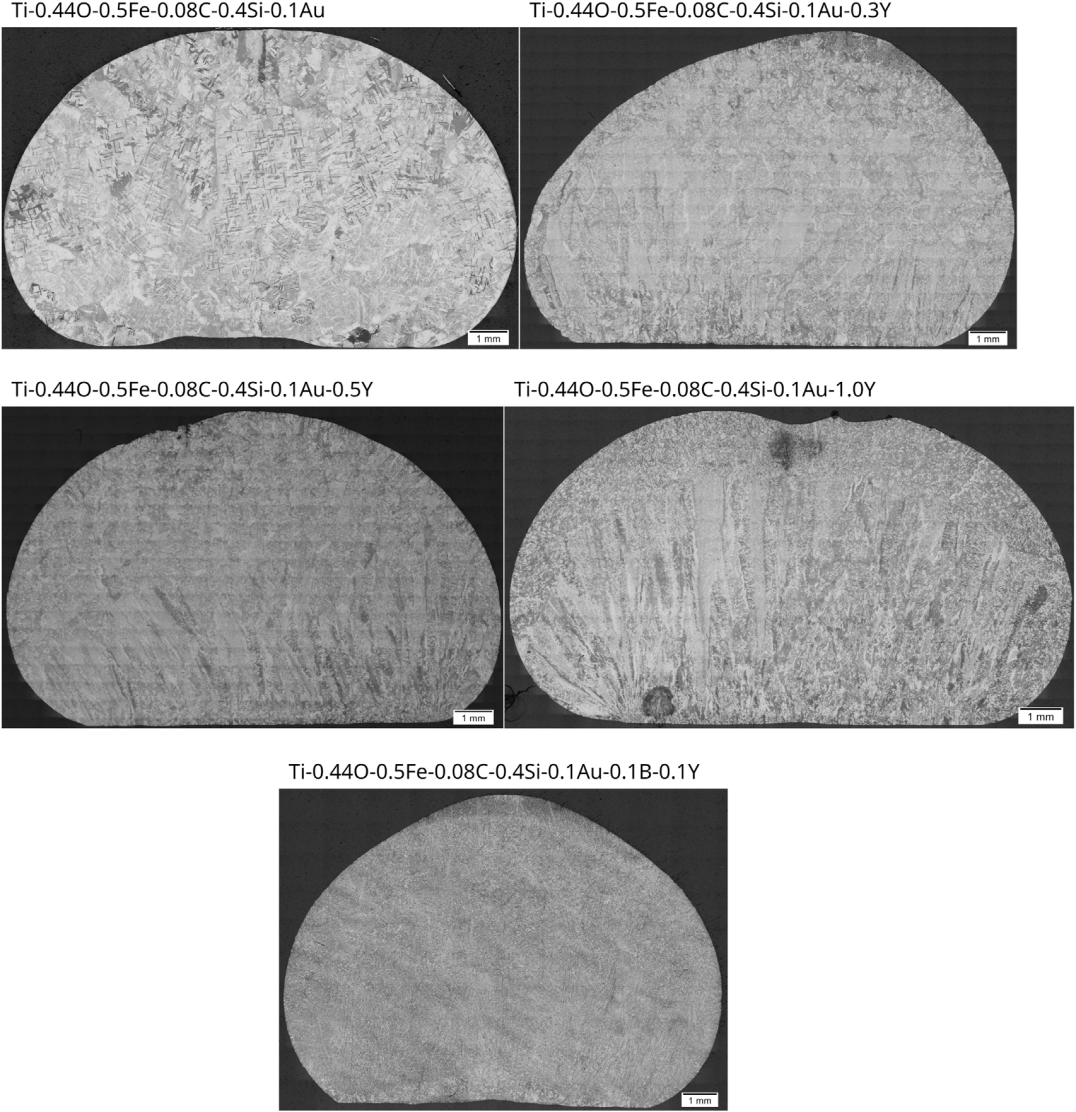
Panorama images of the microstructures of Ti-0.44O-0.5Fe-0.08C-0.4Si-0.1Au (base alloy), Ti-0.44O-0.5Fe-0.08C-0.4Si-0.1Au-(0.3/0.5/1.0)Y, and Ti-0.44O-0.5Fe-0.08C-0.4Si-0.1Au-0.1B-0.1Y in the as-cast state.

Compared to the base alloy, Ti-0.44O-0.5Fe-0.08C-0.4Si-0.1Au, the microstructure was refined with yttrium additions as expected, especially in combination with boron, as can be seen in Figure 8, which shows panorama images of each specimen and the base alloy in the etched state. With the addition of 0.3% Y, the prior β-grain structure was refined significantly. However, columnar prior β-grains are (still) visible on the left side and in the lower part of the sample. Regarding the approximate upper third of the specimen, the identification of prior β-grain boundaries is much more difficult. At the specimen’s tip, the microstructure seems to be very fine with no recognizable prior β-grains boundaries. In addition to β-grain refinement, the length of α-lamellae and the α-colony-size decreased. Nevertheless, some large α-colonies still exist along prior β-grain boundaries, partly having a large width especially for elongated or columnar prior β-grain boundaries. This is also the case in the alloys containing 0.5 Y and 1.0 Y. In terms of the alloy with 0.5% Y, pronounced columnar prior β-grains are visible in the lower half of the specimen (see Figure 8), which seem to have grown epitaxially on the material, which was still solid during melting as being in contact with the water-cooled copper crucible plate. In the upper part, columnar prior β-grains are only visible near the surface area of the specimen. β-grain boundaries are, however, only occasionally identifiable. Very similar features are present in terms of the alloy with 1.0% Y. Significant columnar prior β-grains are visible in the left half of the specimen. α-colonies are also present, partly exhibiting a high width, especially in long, columnar prior β-grains. These columnar prior β-grains end abruptly in the upper region, and a second microstructural area is visible, where prior β-grain boundaries are only sporadically identifiable. Due to the appearance of the α-lamellae, it has to be rather assumed that the prior β-grains within this region are much coarser than in the lower part of the specimen. As a result of these findings, it can be concluded that the propensity for the formation of long columnar prior β-grains originating from the water-cooled copper crucible plate due to epitaxial growth seems to increase with increasing yttrium content.

**FIGURE 9:**
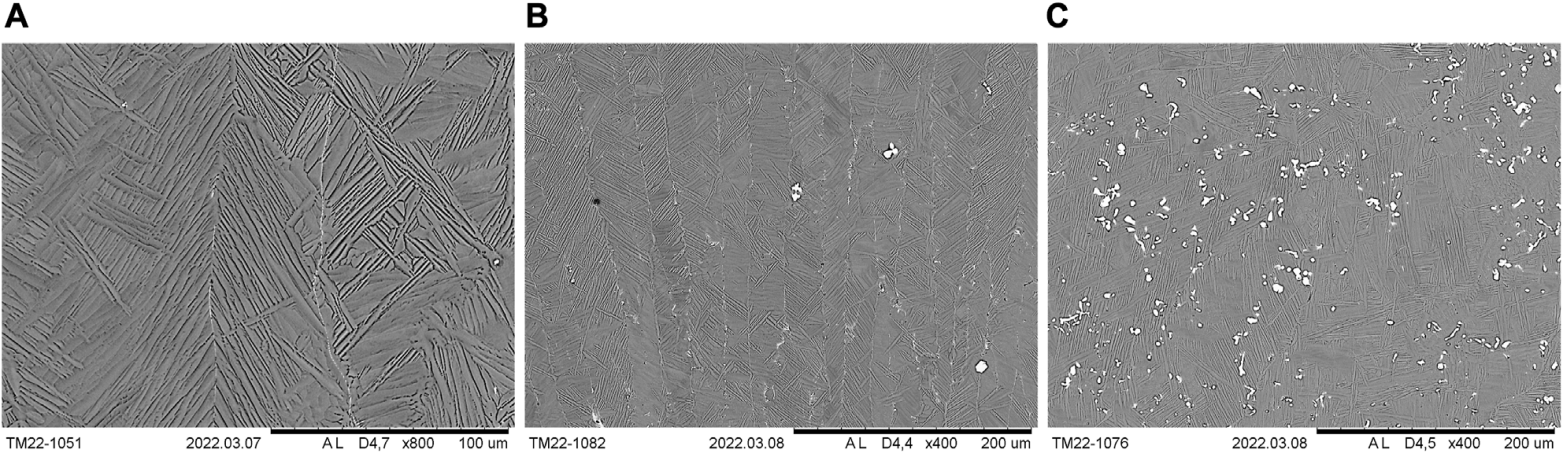
SEM images (BSE detector) of the distribution of yttrium-rich particles in Ti-0.44O-0.5Fe-0.08C-0.4Si-0.1Au-(0.3/0.5/1.0)Y. **(A)** Microstructural area close to the bottom of the specimen of Ti-0.44O-0.5Fe-0.08C-0.4Si-0.1Au-0.3Y, where yttrium-rich particles are present in chain-like structures. **(B)** Smaller chain-like and larger particles or structures in a region at the bottom of the sample of Ti-0.44O-0.5Fe-0.08C-0.4Si-0.1Au-1.0Y. **(C)** Microstructural area of Ti-0.44O-0.5Fe-0.08C-0.4Si-0.1Au-1.0Y containing mostly large yttrium-containing particles or structures.

**FIGURE 10:**
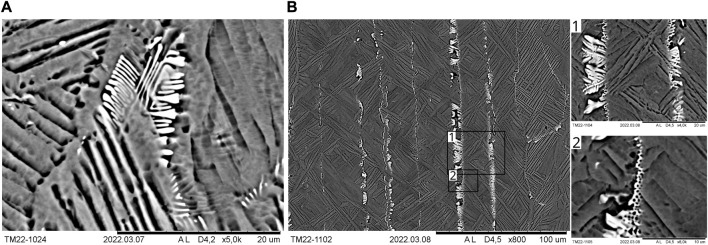
SEM images (BSE detector) of yttrium-rich, more complex structures: **(A)** Ti-0.44O-0.5Fe-0.08C-0.4Si-0.1Au-0.5Y and **(B)** Ti-0.44O-0.5Fe-0.08C-0.4Si-0.1Au-1.0Y.

The SEM analyses of Ti-0.44O-0.5Fe-0.08C-0.4Si-0.1Au-(0.3/0.5/1.0)Y using a BSE detector providing a mass contrast reveal the presence of particles in all specimens, which appear bright in the SEM images and thus contain elements with a higher mass. Local EDS analyses of such particles and of geometrically more complicated structures, which will be described later, indicate increased yttrium and oxygen contents. Since yttrium has a higher mass than the other elements present in the studied alloys except for gold and since no precipitations were identified in previous studies for the base alloy without yttrium, it is expected that all bright particles are yttrium and, possibly, oxygen enriched, as discussed later. Figure 9A shows their occurrence in Ti-0.44O-0.5Fe-0.08C-0.4Si-0.1Au-0.3Y for a spot in the lower half of the specimen close to the bottom. On the left side of this image, bright particles are present chain-like between two α-colonies. Additionally, particles are present within α-lamellae, at α-lamellae and prior β-grain boundaries. On the basis of Figure 9A, it is directly evident that many particles are present even for the yttrium content of only 0.3%. Unsurprisingly, Ti-0.44O-0.5Fe-0.08C-0.4Si-0.1Au-1.0Y contains many particles exhibiting different sizes as shown in Figure 9B, which depicts a region at the bottom of the specimen. Similar to Ti-0.44O-0.5Fe-0.08C-0.4Si-0.1Au-0.3Y, smaller particles are present chain-like within α-lamellae or between α-colonies. Additionally, much larger particles or structures are visible, which are present to a much higher extent in Figure 9C. Aside from these particles, bright, geometrically more complex structures are visible, as exemplarily depicted in Figure 10 for Ti-0.44O-0.5Fe-0.08C-0.4Si-0.1Au-0.5Y (Figure 10A) and Ti-0.44O-0.5Fe-0.08C-0.4Si-0.1Au-1.0Y (Figure 10B). Such structures are, however, also present in Ti-0.44O-0.5Fe-0.08C-0.4Si-0.1Au-0.3Y. As can be seen, these structures differ significantly from the smaller particles described previously. Figure 10A indicates that the depicted structure seems to continue below the specimen's surface or below the visible α-lamellae in the bottom right corner of the image, since its contours are still visible, but with a much lower brightness, due to the interaction volume of the electron beam of the SEM. Consequently, these structures might have a considerable size. Figure 10B shows similar structures being present near chain-like smaller particles, as described previously. Therefore, the latter might not always be isolated particles. In general, such kind of geometrically more complex structures as identified in Ti-0.44O-0.5Fe-0.08C-0.4Si-0.1Au-(0.3/0.5/1.0)Y have already been reported in the literature (Kennedy et al., 2022; Kennedy et al., 2023). This will be discussed in more detail later.

**FIGURE 11:**
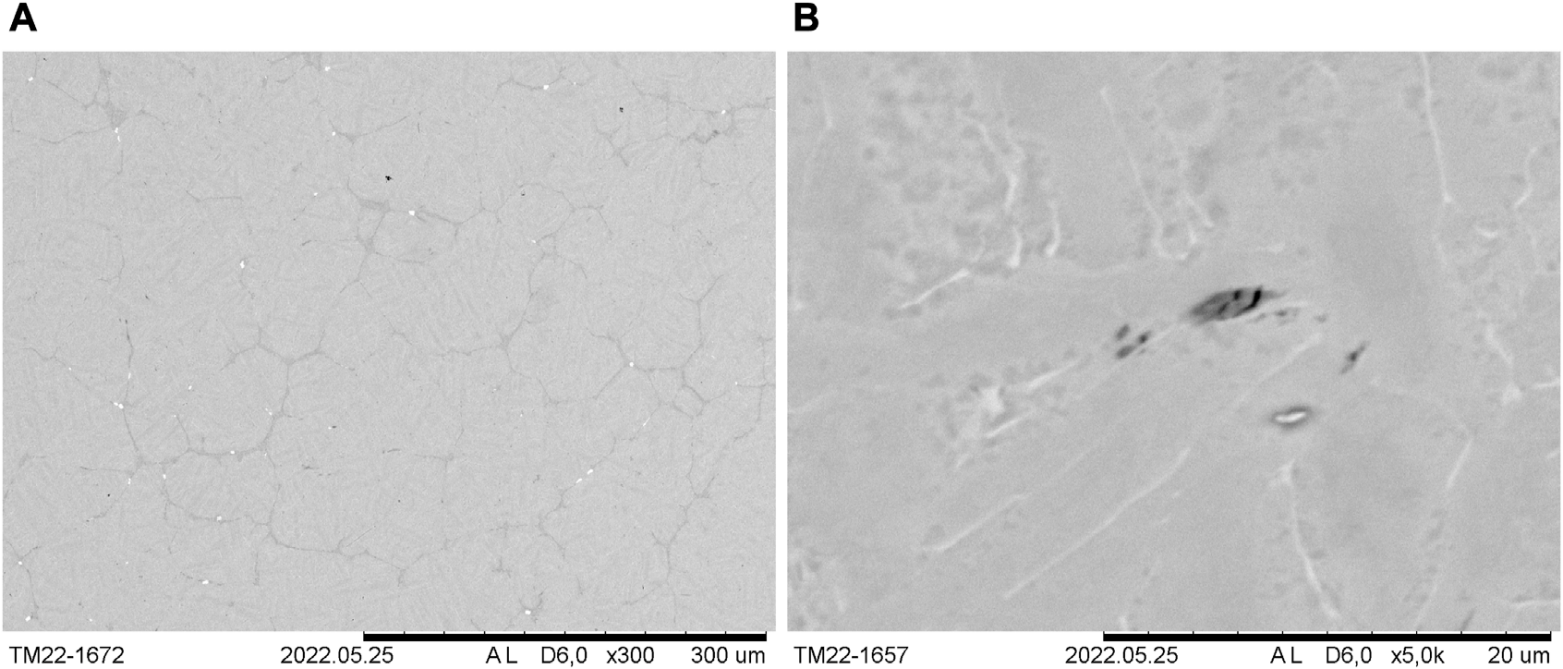
Microstructure (SEM image and BSE detector) of Ti-0.44O-0.5Fe-0.08C-0.4Si-0.1Au-0.1B-0.1Y in the re-polished state. **(A)** Darker gray zones and bright particles enclose equiaxed-like areas. **(B)** Round or needle-like dark spots are present within darker gray areas, suggesting the presence of precipitations.

By contrast, the microstructure of Ti-0.44O-0.5Fe-0.08C-0.4Si-0.1Au-0.1B-0.1Y is significantly different, as can be seen in Figure 8. Prior β-grains or grain boundaries are not identifiable. However, among others, dark gray structures are present, which are elongated in the lower half of the specimen and predominantly equiaxed or elongated in the upper half. The specimen's tip, however, exhibits a much finer solidification microstructure. The SEM analysis of this sample was performed in the polished state (re-polished after etching) in order to study the microstructure using mostly the mass contrast provided by the BSE detector and scarcely the slight topographical contrast, which occurs due to etching. Differences in chemical composition due to microscopic solute segregation can be thus better investigated. Figure 11A shows darker areas and brighter particles. As already described, the latter are supposed to be yttrium rich and, possibly, oxygen enriched. With respect to this particular area, Figure 11A shows the darker gray zones enclose equiaxed-like areas. α- and β-lamellae are also identifiable due to differences in mass contrast and, possibly, a slight residual topographical contrast that is still present due to etching prior to additional polishing. Prior β-grains or grain boundaries are, however, not visible. Additionally, round- or needle-like dark spots are present in the microstructure within darker gray areas, as can be seen in Figure 11B, suggesting the presence of precipitations.

The results of the Vickers hardness tests are summarized in Table 3. As can be seen, the base alloy Ti-0.44O-0.5Fe-0.08C-0.4Si-0.1Au exhibits the highest hardness of approximately 312 HV10. A monotonic decrease of hardness with increasing yttrium content is not present. Nevertheless, the 1.0% yttrium containing alloy exhibits the lowest hardness of approximately 269 HV10.

**TABLE 3:** Vickers hardness test results for Ti-0.44O-0.5Fe-0.08C-0.4Si-0.1Au-(0.3/0.5/1.0)Y, Ti-0.44O-0.5Fe-0.08C-0.4Si-0.1Au-0.1B-0.1Y, and the base alloy without yttrium.

	0Y	0.3Y	0.5Y	1.0Y	0.1Y 0.1B
Hardness [HV10]	312^a^ ^,^ ^b^	293	297	269	297
Standard deviation [HV10]	13.1^a^ ^,^ ^b^	4.4	7.7	11.6	3.7

^a^ Specimen taken from Haase et al. (2023).

^b^ Only four indentations were measurable.

#### 3.2.2 Phase analysis

**FIGURE 12:**
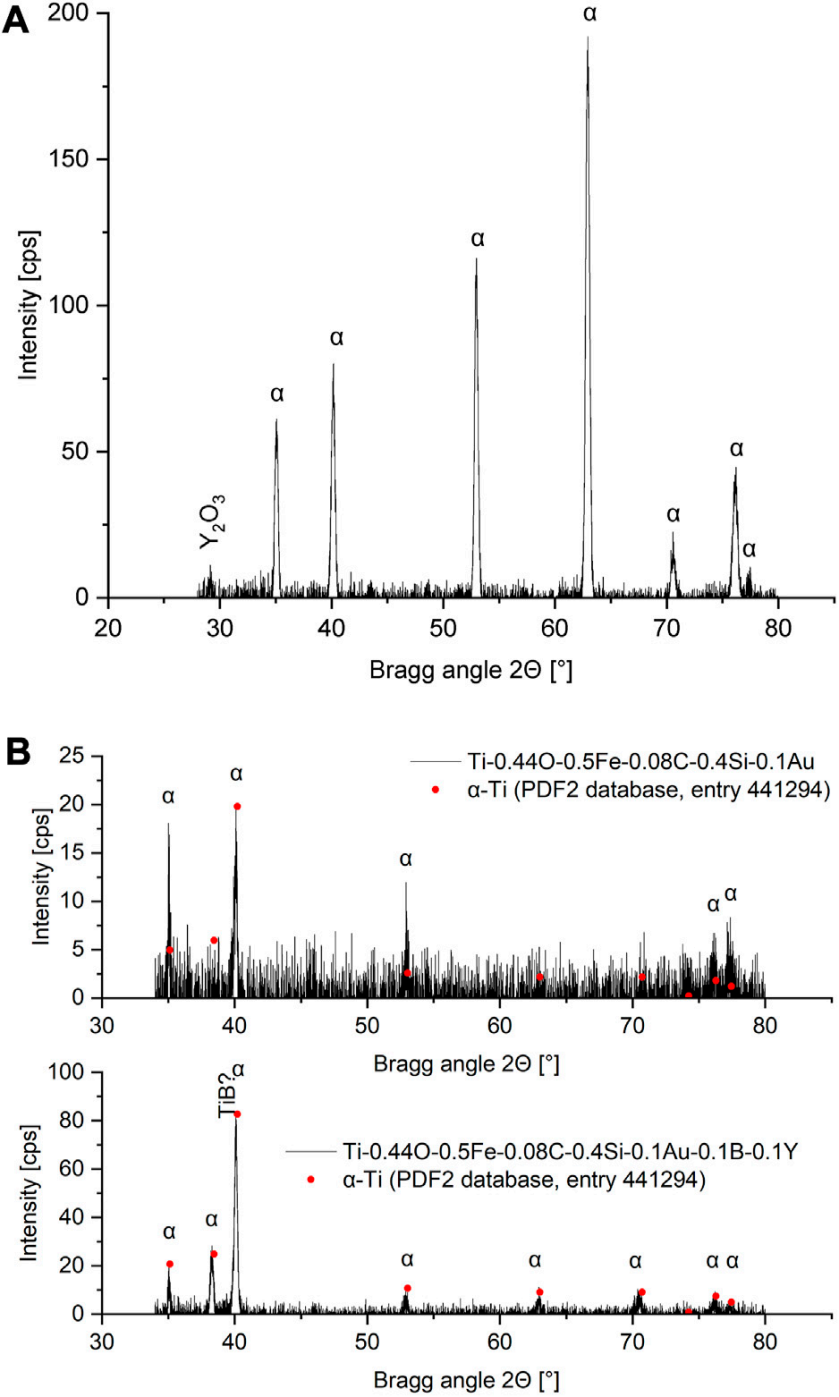
Diffraction results obtained for three studied alloys with assigned phases. **(A)** Ti-0.44O-0.5Fe-0.08C-0.4Si-0.1Au-1.0Y. **(B)** Comparison of Ti-0.44O-0.5Fe-0.08C-0.4Si-0.1Au (base alloy) with Ti-0.44O-0.5Fe-0.08C-0.4Si-0.1Au-0.1B-0.1Y. Included are peak positions and rescaled intensities for pure α-titanium, obtained from the PDF2 database.

Figure 12A shows the diffractogram of Ti-0.44O-0.5Fe-0.08C-0.4Si-0.1Au-1.0Y together with assigned phases. Only distinct peaks are indicated, whereas other possible peaks that are characterized only by increased background radiation are not considered. The first peak at an angle of approximately 29.15°, as can be seen, is also mostly characterized by increased background radiation. However, this peak exhibits a very good agreement with the main peak of the phase Y_2_O_3_. In addition, it can be concluded that the analyzed volume of the sample during XRD measurement is characterized by substantial texture since, among others, the { $10\bar{1}1$ }_α_-peak at an angle of approximately 40.15° is, in contrast to a texture free sample, not the main peak in the diffractogram. This could be caused by the point focus of the X-ray tube in combination with the small pinhole collimator used during XRD measurements; this setup projects only a small point as the radiated area on the specimen's surface, which gets elliptically distorted at small Bragg angles. As a result, the radiated area or volume is rather small, so that possibly only a small number of α-lamellae or α-colonies diffracted the radiation. During this measurement, the elliptical distortion occurred in the horizontal direction of the specimens’ microstructure depicted in Figure 8.

The diffractograms of the base alloy Ti-0.44O-0.5Fe-0.08C-0.4Si-0.1Au and Ti-0.44O-0.5Fe-0.08C-0.4Si-0.1Au-0.1B-0.1Y are shown in Figure 12B. In both cases, the line focus of the X-ray tube was used for measuring, leading to a much larger irradiated surface area. As can be seen in the base alloy, the signal-to-noise ratio is quite low, making the background subtraction (as performed) difficult. Aside from a small peak of the embedding material, characterized by an increased background radiation, and therefore not visible in Figure 12B, only α-titanium can be identified. Moreover, according to the differences between the ideal and measured peak intensity ratios, the material should be textured considerably. By contrast, the diffractogram of Ti-0.44O-0.5Fe-0.08C-0.4Si-0.1Au-0.1B-0.1Y exhibits a much better signal-to-noise ratio and α-peak intensity ratios close to the ideal values, which suggest a comparable low texture and more isotropic properties. Again, a peak of the embedding material leads to an increase in background radiation that is not visible in Figure 12B. Additionally, the main α-peak at an angle of approximately 40.1° is slightly asymmetric toward lower angles, which could suggest an overlap of the α peak flank with a TiB peak. Other possible TiB peaks cannot be identified. Considerable peaks of this phase are, however, not expected due to the low volume fraction.

## 4 Discussion

### 4.1 LPBF of CP titanium grade 1

The Doehlert design used within this study enabled the successfull determination of suitable volume process parameters for LPBF printing of small cubical specimens in order to achieve low residual porosity in the as-printed state. In addition, a process window seemed to be established, so that a suitable combination of laser power, hatch distance, and scanning speed could be selected depending on other variables such as the processing time. However, as already described, the calculated equation cannot accurately describe the porosity in all sets of process parameters falling within the studied experimental domain. In the described case, substantial porosity due to the lack of fusion, caused by very low energy density, is not represented. It is therefore necessary to verify the usability of derived process parameters. A better total approximation might be achieved if the experimental domain is kept smaller. Regarding the present study, the laser power, hatch distance, and scanning speed varied considerably, since several different process parameters for LPBF processing of CP titanium were used or studied in the existing literature (Gu et al., 2012; Li et al., 2013; Attar et al., 2014; Attar et al., 2017; Wysocki et al., 2017; Na et al., 2018; Wang et al., 2019). However, the established process parameters and the obtained equation are only usable for small samples, such as, dental implants. The larger specimens exhibited a low surface quality. This shows that other parameters such as gas flow, part size, or further parameters affecting the scanning pattern that are not covered by the experimental design could have a significant effect on the obtained results. In order to find a suitable set of processing parameters that optimize two responses at once, such as porosity and surface quality, other optimization methods might be more suitable (Gajera et al., 2022). Additionally, it was shown that two very different energy densities during LPBF printing lead to very low porosities. Similar results have already been reported by Li et al. (2013). This suggests that the energy density is ideally not suitable to summarize a combination of hatch distance, layer thickness, laser power, and scanning speed in order to compare the process parameter sets in view of relative density, which is often employed in the literature. Limitations during the use of energy density as a parameter have already been described in the literature (Bertoli et al., 2017).

The obtained mechanical properties of the LPBF printed material are exceptional with high strength and high ductility. The mechanical properties for LPBF-processed CP titanium described in the literature (Li et al., 2013; Attar et al., 2014; Wysocki et al., 2017; Na et al., 2018; Wang et al., 2019) are partly similar and partly dissimilar from the results of the present study. However, a direct comparison is sometimes difficult due to the different tensile specimens or mechanical tests. In terms of the present study, the higher YTS and UTS of the LPBF-processed material when compared to the commercial recrystallized rod material can be explained, on the one hand, by the higher initial oxygen and iron content of the powder when compared to the rod material, therefore leading to a higher solid solution strengthening effect. This might be further increased by the pickup of considerable amounts of oxygen and nitrogen during LPBF processing as described in Section 3.1.2. However, the exact oxygen and nitrogen contents within the measuring section of the tensile specimens are unknown, since they are located at higher building heights than the samples that were used for elemental analysis and probably printed under a more pure processing atmosphere. On the other hand, the LPBF material exhibits a finer microstructure. Consequently, a higher strength is expected according to the well-known Hall–Petch relationship. The optimized LPBF volume process parameters and mechanical properties obtained in the present study might render the production of small components directly usable without further heat treatment or hot isostatic pressing, if only static loads are applied. However, for applications with dynamic loads, a post-processing treatment via hot isostatic pressing would be necessary, since residual porosity and, especially, single larger pores that have been observed in our CT measurements can have a substantial impact on fatigue life (DebRoy et al., 2018). Despite this, defects do remain in the part after post-processing and influence the mechanical properties (He et al., 2022). Consequently, fatigue life of LPBF-processed implants should be predicted before usage using appropriate models, which distinguishes between defects being present at the surface or in the interior of components, as was described by He et al. (2022).

According to the presented XRD results, the LPBF-processed material should possess anisotropic mechanical properties. However, these do not seem to be very pronounced at least for the analyzed specimen orientations on the substrate plate. Notably, the YTS and UTS of specimens 1 and 2 with the tensile testing direction being parallel to the gas flow direction seem to be lower than that of the other specimens, whereas the modulus of elasticity seems to be slightly higher. In general, the modulus of elasticity of the hexagonal unit cell of α-titanium significantly depends on the loading direction (Lütjering and Williams, 2007). Consequently, a possible interpretation would be that α-grains are oriented in such a way that the stiffness is increased in specimens 1 and 2. However, tensile tests are not a reliable method to measure the modulus of elasticity precisely (DIN Deutsches Institut für Normung e.V, 2017). Moreover, as already indicated, the number of specimens tested for each orientation is not sufficient to allow a reliable statement on a possible orientation dependence of mechanical properties. Consequently, the difference in elasticity might not be statistically significant. By contrast, the lower YTS and UTS might be caused by defects present in these samples because long edges parallel to the gas flow direction are prone for defect formation, since the laser beam moves considerably with the gas flow direction during border processing. To summarize, further investigations are required to characterize a possible orientation dependence of mechanical properties with respect to crystallographic texture.

### 4.2 Alloy development

#### 4.2.1 Solidification and microstructure

The results presented show that yttrium and boron additions are beneficial in order to refine the microstructure of Ti-0.44O-0.5Fe-0.08C-0.4Si-0.1Au as expected. In general, a refinement effect of yttrium (added in the elemental state or as Y_2_O_3_) either on β- or α-grain size has already been described in the literature (Nordin et al., 1987; Cui et al., 2002; Zhang et al., 2020b; Wang et al., 2021; Kennedy et al., 2022) as well as refinement effects of boron on, among others, β-grains or α-colonies during conventional casting and additive manufacturing (Tamirisakandala et al., 2005; Bermingham et al., 2008; Bermingham et al., 2015; Mantri et al., 2017; Xue et al., 2019; Zhang et al., 2019). Microstructure refinement was consequently expected. However, β-refinement seems to occur only for certain yttrium content. Although the width of β-grains near the bottom of the specimens decreases with increasing yttrium content, as seen in Figure 8, prior β-grains growing epitaxially from the unmolten material at the water-cooled copper crucible plate of the arc furnace remain columnar and even seem to be much more elongated in terms of the alloy containing 1.0% Y. Additionally, for this alloy, it can be seen that the width increases considerably in solidification or columnar direction, probably indicating an outgrowth of crystallographic less favorably oriented β-grains. Consequently, it is expected that these columnar grains exhibit a texture due to preferential growth of the β-crystal lattice along certain crystallographic directions (Nordin et al., 1987; DebRoy et al., 2018), so that substantial changes in texture and thus much more isotropic mechanical properties are not expected. This could be supported by the XRD measurements performed on this sample, as already described. Similar results with refined but still columnar prior β-grains exhibiting a preferential, yet weaker texture have already been reported by Kennedy et al. (2022) for yttrium-modified Ti-6Al-4V during wire-arc additive manufacturing. However, Zhang et al. (2020b) reported similar columnar prior β-grains to be present only if Y_2_O_3_ powder is added to Ti-6Al-4V, whereas elemental yttrium led to a substantial refinement and equiaxed prior β-grains. Consequently, the present study suggests to keep the yttrium content low, also due to decreasing hardness and increasing amount of particles with increasing yttrium content, as discussed in the following paragraph. Ti-0.44O-0.5Fe-0.08C-0.4Si-0.1Au-(0.3/0.5/1.0)Y are ideally not suitable to obtain good mechanical properties with lower anisotropy for medical applications.

Regarding Ti-0.44O-0.5Fe-0.08C-0.4Si-0.1Au-0.1B-0.1Y, the darker gray areas described previously are regarded as boron-enriched zones formed due to boron segregation within such as intercellular or interdendritic regions during solidification. Boron segregation during solidification of this alloy is expected, since boron exhibits a very high growth restriction factor and partitions strongly into the liquid (Zhang et al., 2020a). Consequently, elongated cells or dendrites are present in the lower half of the specimen, indicating either a cellular or columnar dendritic solidification. Columnar prior β-grains are therefore expected within this region. In most cases, however, the solidification microstructure cannot be determined exactly, since the segregation zones are not very pronounced. By contrast, the equiaxed-like areas enclosed by these segregations such as visible in Figure 11A are regarded as equiaxed prior β-grains. As described by Tamirisakandala et al. (2005) and Tamirisakandala and Miracle (2010), during solidification and growth of β-grains, boron is rejected into the liquid, and once the eutectic temperature is reached, the boron-enriched liquid between β-grains transforms via a eutectic transformation in β-phase and TiB. Consequently, after complete solidification, β-grains are surrounded by TiB (Tamirisakandala et al., 2005; Tamirisakandala and Miracle, 2010). Thus, the microstructure shown in Figure 11A strongly suggests the presence of equiaxed prior β-grains. These are desired in order to obtain (more) isotropic mechanical properties (DebRoy et al., 2018; StJohn et al., 2018; Zhang et al., 2020a). Consequently, it is expected that the much darker round- or needle-like spots observed in Figure 11B are TiB precipitations. However, due to their small size, the EDS analysis was not possible for further characterization. Nevertheless, the qualitative diffraction analysis performed for this alloy could suggest the presence of a TiB peak, as already described. Verification of TiB precipitations by XRD analysis is expected to be aggravated due to the comparable low volume fraction of this phase.

Regarding Ti-0.44O-0.5Fe-0.08C-0.4Si-0.1Au-(0.3/0.5/1.0)Y and Ti-0.44O-0.5Fe-0.08C-0.4Si-0.1Au-0.1B-0.1Y, it can be assumed that the bright particles visible in the SEM images are Y_2_O_3_ according to the EDS analyses performed and according to the results described in the literature (Cui et al., 2002; Zhang et al., 2020b; Kennedy et al., 2022). Furthermore, the small chain-like particles seem to be present in intercellular or interdendritic regions as can be seen in Figure 9, which is consistent with the observations of Kennedy et al. (2022), thus mirroring the solidification microstructure being either cellular or columnar dendritic within this region. Bigger particles in Ti-0.44O-0.5Fe-0.08C-0.4Si-0.1Au-1.0Y as seen in Figure 9C seem to, however, be more randomly distributed. Since a cellular or columnar dendritic solidification leads to columnar prior β-grains (DebRoy et al., 2018), the observed columnar grains in the lower part of the specimens are consistent with this observation. The formation of Y_2_O_3_ in the microstructure of yttrium-containing titanium alloys is, thereby, extensively studied in the literature. The formation of Y_2_O_3_ is related to oxidation either before or during solidification with oxygen taken from the liquid (Tian et al., 2006; Zhang et al., 2020b; Kennedy et al., 2022). The hardness measurements performed in the present study can support this explanation; since oxygen leads to a substantial solid solution strengthening effect (Lütjering and Williams, 2007), which has also been investigated and established for similar titanium alloys compared to those investigated in the present study including Ti-0.44O-0.5Fe-0.08C-0.4Si-0.1Au (Haase et al., 2020), a significant hardness decrease would be expected if Y_2_O_3_ is formed at the expense of dissolved oxygen. This significant decrease is present in Ti-0.44O-0.5Fe-0.08C-0.4Si-0.1Au-1.0Y, exhibiting the lowest hardness of all studied alloys. However, a monotonic hardness decrease with increasing yttrium content would also be expected in this case, which, as already described, is not observed for Ti-0.44O-0.5Fe-0.08C-0.4Si-0.1Au-(0.3/0.5/1.0)Y. Maybe other parameters affecting hardness do also play a role such as precipitation hardening (partly) compensating the loss in solid solution strengthening. Taking this and the results and explanations of Kennedy et al. (2022) into account, it could be possible in terms of the observed chain-like small particles that elemental yttrium is enriched in the liquid during solidification within intercellular or interdendritic regions and transformed to Y_2_O_3_ at some point by taking oxygen from the alloy, which leads to a softening of the matrix. This point in time when Y_2_O_3_ was formed cannot be determined. According to solidification calculations performed by Kennedy et al. (2022), direct formation of Y_2_O_3_ at the solid–liquid interface at the later stages of solidification might be possible. In regard to this, increased oxygen content in the present alloys might have an effect on the formation of Y_2_O_3_ (Kennedy et al., 2022). On the other hand, due to decreasing solid solubility of yttrium in β-Ti with decreasing temperature and very low solubility in α-Ti according to the binary Ti-Y phase diagram (Murray, 1986), elemental yttrium precipitation might have occurred after complete solidification out of the β- or α-phase during cooling within the intercellular or interdendritic regions with subsequent formation of Y_2_O_3_. Furthermore, in contrast to both these possible explanations, the results of the present authors' working group on the rare earth element lanthanum in titanium allow an additional assumption on the formation of Y_2_O_3_. During the metallographic analysis of lanthanum-containing Ti-6Al-4V, La_2_O_3_ particles were identified by XRD experiments to be present within the specimen's surface (low penetration depth of the X-ray radiation) (Siemers et al., 2007). However, diffraction experiments performed in transmission using high-energy synchrotron radiation in different lanthanum-containing titanium alloys that also contain different oxygen contents have proved that no lanthanum oxide is present within the bulk material (Siemers et al., 2007). Instead, elemental/metallic lanthanum particles were formed, which are already present in the as-cast state (and an additional phase, which could not be determined) (Siemers et al., 2007). Thus, the possibility that substantial formation of Y_2_O_3_ does not happen until, for example, specimen preparation cannot be neglected. Therefore, further investigations with *in situ* XRD experiments using high-energy synchrotron radiation are necessary to determine the stage at which the formation of Y_2_O_3_ takes place during melting or solidification.

By contrast, the bigger particles observed in Ti-0.44O-0.5Fe-0.08C-0.4Si-0.1Au-1.0Y, as is evident in Figure 9C, do not mirror the solidification microstructure. Thus, it could be possible that these particles are already transformed to Y_2_O_3_ before the onset of solidification and have become embedded in the microstructure during solidification as has been described by Zhang et al. (2020b). In regard to this, the particles observed in the liquid during some or—as in the case of Ti-0.44O-0.5Fe-0.08C-0.4Si-0.1Au-1.0Y—each remelting step that is described in the Results section might have been Y_2_O_3_, which dissolved only slowly or not at all during remelting. Moreover, as already described, during the last remelting of Ti-0.44O-0.5Fe-0.08C-0.4Si-0.1Au-0.5Y, solidification seemed to have started at such a particle within the melt in addition to the main solidification front originating near the water-cooled copper plate. The possibility that Y_2_O_3_ acts as solidification nuclei for the β-phase is comprehensively discussed in the literature (Nordin et al., 1987; Zhang et al., 2020b; Wang et al., 2021; Kennedy et al., 2022), and the (possible) orientation relationships between prior β-grains and Y_2_O_3_ have been proposed or discussed (Zhang et al., 2020b; Kennedy et al., 2022). A nucleant effect certainly requires Y_2_O_3_ to be present in the liquid during solidification, such as due to remelting of previous Y_2_O_3_-containing layers during additive manufacturing (Zhang et al., 2020b). However, this requires the formation of Y_2_O_3_ in the microstructure during solidification or cooling, which, as already described, has to be further investigated.

Regarding the geometrically more complex structures being present in Ti-0.44O-0.5Fe-0.08C-0.4Si-0.1Au-(0.3/0.5/1.0)Y, similar structures have already been reported in the literature (Kennedy et al., 2022; Kennedy et al., 2023) and been described as having a similar shape to structures formed during irregular eutectic solidification (Kennedy et al., 2022). The present results shown in Figure 10 seem to support this assumption of eutectic solidification. However, a possible eutectoid reaction with β-Ti transforming to α-Ti and α-Y according to the binary Ti-Y phase diagram (Murray, 1986) has to be considered as well.

As a result, further investigations are necessary for yttrium-containing titanium alloys to investigate the formation of Y_2_O_3_ particles and of geometrically more complex structures in the microstructure. Further possible effects of elemental yttrium as being surface active (Tian et al., 2006) have to be taken into account as well.

#### 4.2.2 Alloy and texture assessments

According to the XRD results presented in Figure 12B, it is expected that Ti-0.44O-0.5Fe-0.08C-0.4Si-0.1Au-0.1B-0.1Y exhibits a comparable low quantitative texture and therefore more isotropic mechanical properties. This texture reduction when compared to Ti-0.44O-0.5Fe-0.08C-0.4Si-0.1Au could be linked with finer β- and α-colonies. This relationship and underlying limitations have already (but more briefly) been described for Ti-0.44O-0.5Fe-0.08C-0.4Si-0.1Au and silicon-containing variants of this alloy (Haase et al., 2023). If α-colonies are present within the microstructure, these colonies and not individual α-lamellae are important for texture analysis as α-lamellae within a colony exhibit the same orientation toward the parent β-grain (Lütjering and Williams, 2007). Therefore, a decrease in colony size or complete suppression of their formation due to alloying elements could lead to more α-orientations, to a certain extend. However, this effect is limited by the Burgers orientation relationship, since it limits the possible orientations that α-lamellae can exhibit in a parent β-grain (Lütjering and Williams, 2007). The amount or size and crystallographic orientation of the β-grains formed during solidification of conventionally melted or LPBF-processed material are thus important, since the Burgers orientation relationship predetermines the α-orientations after allotropic transformation on the basis of the crystal orientation of the parent β-grain (Barriobero-Vila et al., 2018). Long, columnar prior β-grains being present in Ti-0.44O-0.5Fe-0.08C-0.4Si-0.1Au-1.0Y are thus detrimental as already described, since the crystal lattice of columnar prior β-grains formed for example during additive manufacturing exhibits a preferred orientation toward the temperature gradient or the building direction (Barriobero-Vila et al., 2018; DebRoy et al., 2018). Therefore, the obtained equiaxed prior β-grains on Ti-0.44O-0.5Fe-0.08C-0.4Si-0.1Au-0.1B-0.1Y are promising. In addition, refined β-grains lead to refined α-lamellae during allotropic transformation (Cui et al., 2002). Further explanations for texture reduction might be possible on the basis of Y_2_O_3_ or TiB particles. Wang et al. (2021) investigated and discussed the possibility of Y_2_O_3_ acting as nucleant for the formation of α-grains during allotropic transformation, consequently leading to a refined microstructure. In addition, the pinning effect of Y_2_O_3_ on (prior) β-grains was discussed (Cui et al., 2002; Ueda et al., 2013; Zhang et al., 2020b; Wang et al., 2021). Furthermore, the nucleation of α-grains on TiB particles was investigated and described in several publications, as summarized by Singh and Ramamurty (2021).

## 5 Conclusion

In the present study, a suitable combination of laser power, scanning speed, and hatch distance was determined via a design of experiments for LPBF printing of CP titanium grade 1 powder to achieve low residual porosity in small samples. Using these optimized parameters and a rotating scanning pattern, a high relative density of approximately 99.9% was achieved in the as-printed state according to optical analysis. Moreover, tensile specimens processed with these settings exhibit a very high average YTS and UTS of approximately 663 and 747 N/mm^2^, respectively, together with an average ductility of approximately 24% (considering representative specimens only) in the as-printed milled state. In addition, a process window was established, which assists the finding of suitable volume process parameters to achieve high relative density. Further investigations are necessary for a possible orientation dependence of the mechanical properties of CP titanium parts manufactured with the obtained process parameters, as suggested by XRD measurements. In addition, on the basis of the described limitations of the derived process parameters and process window, further validation experiments are necessary to prove their applicability. This is especially the case for larger parts and parts with more complex geometric features, which might require adaptions in volume or border processing. Moreover, the influence of other parameters not covered by the present experimental design but affecting part quality, such as gas flow velocity, should be considered as well.

On the basis of conventionally melted alloys, a new titanium alloy, Ti-0.44O-0.5Fe-0.08C-0.4Si-0.1Au-0.1B-0.1Y, is proposed as a potential candidate for LPBF processing of medical implants with lower anisotropy and higher biocompatibility than the established conventional (α+β) biomedical titanium alloys. The new alloy exhibits refined microstructure and improved solidification due to the use of oxygen, carbon, iron, silicon, yttrium, and boron as its alloying elements, that provide, among others, growth restriction. Nevertheless, further investigations are required for this alloy in terms of microstructure, texture, and especially mechanical properties after LPBF processing and possible post-processing treatment, since sufficient mechanical properties are the key requirement for a biocompatible titanium alloy. Moreover, the present study complements the current understanding of microstructure formation in yttrium-containing titanium alloys and outlines current frontiers, which require further investigation. In regard to this, the formation of Y_2_O_3_ in yttrium-containing titanium alloys has to be explored further. This should be performed for conventionally processed and additively manufactured alloys in order to take very different characteristics of the associated processes, such as the cooling rate, temperature gradient, and solidification, into account.

## Data Availability

The raw data supporting the conclusions of this article will be made available by the authors, without undue reservation.

## References

[B1] ASTM International (2013a). ASTM B348-13. Standard specification for titanium and titanium alloy bars and billets. West Conshohocken, PA: ASTM International. 10.1520/B0348-13

[B2] ASTM International (2013b). ASTM F67-13. Standard specification for unalloyed titanium, for surgical implant applications (UNS R50250, UNS R50400, UNS R50550, UNS R50700). West Conshohocken, PA: ASTM International. 10.1520/F0067-13

[B3] Attar H. Calin M. Zhang L. C. Scudino S. Eckert J. (2014). Manufacture by selective laser melting and mechanical behavior of commercially pure titanium. Mat. Sci. Eng. A 593, 170–177. 10.1016/j.msea.2013.11.038

[B4] Attar H. Ehtemam-Haghighi S. Kent D. Wu X. Dargusch M. S. (2017). Comparative study of commercially pure titanium produced by laser engineered net shaping, selective laser melting and casting processes. Mat. Sci. Eng. A 705, 385–393. 10.1016/j.msea.2017.08.103

[B5] Bahl S. Raj S. Vanamali S. Suwas S. Chatterjee K. (2014). Effect of boron addition and processing of Ti–6Al–4V on corrosion behaviour and biocompatibility. Mat. Technol. 29, B64–B68. 10.1179/1753555713Y.0000000118

[B6] Barriobero-Vila P. Gussone J. Stark A. Schell N. Haubrich J. Requena G. (2018). Peritectic titanium alloys for 3D printing. Nat. Commun. 9, 3426. 10.1038/s41467-018-05819-9 30143641 PMC6109080

[B7] Bermingham M. J. Kent D. Zhan H. StJohn D. H. Dargusch M. S. (2015). Controlling the microstructure and properties of wire arc additive manufactured Ti–6Al–4V with trace boron additions. Acta Mater 91, 289–303. 10.1016/j.actamat.2015.03.035

[B8] Bermingham M. J. McDonald S. D. Nogita K. St. John D. H. Dargusch M. S. (2008). Effects of boron on microstructure in cast titanium alloys. Scr. Mat. 59, 538–541. 10.1016/j.scriptamat.2008.05.002

[B9] Bertoli U. S. Wolfer A. J. Matthews M. J. Delplanque J.-P. R. Schoenung J. M. (2017). On the limitations of volumetric energy density as a design parameter for selective laser melting. Mat. Des. 113, 331–340. 10.1016/j.matdes.2016.10.037

[B10] Chen L.-Y. Liang S.-X. Liu Y. Zhang L.-C. (2021). Additive manufacturing of metallic lattice structures: unconstrained design, accurate fabrication, fascinated performances, and challenges. Mat. Sci. Eng. R. Rep. 146, 100648. 10.1016/j.mser.2021.100648

[B11] Cui W. F. Liu C. M. Zhou L. Luo G. Z. (2002). Characteristics of microstructures and second-phase particles in Y-bearing Ti-1100 alloy. Mat. Sci. Eng. A 323, 192–197. 10.1016/S0921-5093(01)01362-4

[B12] Davoodi E. Montazerian H. Mirhakimi A. S. Zhianmanesh M. Ibhadode O. Shahabad S. I. (2022). Additively manufactured metallic biomaterials. Bioact. Mat. 15, 214–249. 10.1016/j.bioactmat.2021.12.027 PMC8941217 35386359

[B13] DebRoy T. Wei H. L. Zuback J. S. Mukherjee T. Elmer J. W. Milewski J. O. (2018). Additive manufacturing of metallic components – process, structure and properties. Prog. Mat. Sci. 92, 112–224. 10.1016/j.pmatsci.2017.10.001

[B14] DIN Deutsches Institut für Normung e.V. (2016). DIN 50125:2016-12, Testing of metallic materials – tensile test pieces. Berlin: Beuth Verlag GmbH. 10.31030/2577390

[B15] DIN Deutsches Institut für Normung e.V. (2018). DIN EN ISO 6507-1:2018-07, Metallic materials – Vickers hardness test – Part 1: Test method (ISO 6507-1:2018); German version EN ISO 6507-1:2018. Berlin: Beuth Verlag GmbH. 10.31030/2778746

[B16] DIN Deutsches Institut für Normung e.V. (2017). DIN EN ISO 6892-1:2017-02, Metallic materials – tensile testing – Part 1: Method of test at room temperature (ISO 6892-1:2016); German version EN ISO 6892-1:2016. Berlin: Beuth Verlag GmbH). 10.31030/2384831

[B17] Doehlert D. H. (1970). Uniform shell designs. J. R. Stat. Soc. Ser. C Appl. Stat. 19, 231–239. 10.2307/2346327

[B18] Ebel T. Blawert C. Willumeit R. Luthringer B. J. C. Ferri O. M. Feyerabend F. (2011). Ti-6Al-4V-0.5B-A modified alloy for implants produced by metal injection molding. Adv. Eng. Mat. 13, B440–B453. 10.1002/adem.201180017

[B19] Ferreira S. L. C. dos Santos W. N. L. Quintella C. M. Neto B. B. Bosque-Sendra J. M. (2004). Doehlert matrix: A chemometric tool for analytical chemistry–review. Talanta 63, 1061–1067. 10.1016/j.talanta.2004.01.015 18969534

[B20] Feyerabend F. Fischer J. Holtz J. Witte F. Willumeit R. Drücker H. (2010). Evaluation of short-term effects of rare earth and other elements used in magnesium alloys on primary cells and cell lines. Acta. Biomater. 6, 1834–1842. 10.1016/j.actbio.2009.09.024 19800429

[B21] Froes F. H. (2018). “Titanium for medical and dental applications – An introduction,” in Titanium in medical and dental applications. Editors Froes F. H. Qian M. (Duxford, United Kingdom: Woodhead Publishing), 3–21. 10.1016/B978-0-12-812456-7.00001-9

[B22] Gajera H. Djavanroodi F. Kumari S. Abhishek K. Bandhu D. Saxena K. K. (2022). Optimization of selective laser melting parameter for Invar material by using JAYA algorithm: comparison with TLBO, GA and JAYA. Materials 15, 8092. 10.3390/ma15228092 36431576 PMC9693503

[B23] García Campaña A. M. Cuadros Rodríguez L. Lupiañez González A. Alés Barrero F. Román Ceba M. (1997). Sequential response surface methodology for multioptimization in analytical chemistry with three-variable Doehlert designs. Anal. Chim. Acta 348, 237–246. 10.1016/S0003-2670(97)00155-4

[B24] Geetha M. Singh A. K. Asokamani R. Gogia A. K. (2009). Ti based biomaterials, the ultimate choice for orthopaedic implants – a review. Prog. Mat. Sci. 54, 397–425. 10.1016/j.pmatsci.2008.06.004

[B25] Gu D. Hagedorn Y.-C. Meiners W. Meng G. Batista R. J. S. Wissenbach K. (2012). Densification behavior, microstructure evolution, and wear performance of selective laser melting processed commercially pure titanium. Acta Mater. 60, 3849–3860. 10.1016/j.actamat.2012.04.006

[B26] Gubbi P. Wojtisek T. (2018). “The role of titanium in implant dentistry,” in Titanium in medical and dental applications. Editors Froes F. H. Qian M. (Duxford, United Kingdom: Woodhead Publishing), 505–529. 10.1016/B978-0-12-812456-7.00023-8

[B27] Haase F. Siemers C. Goldapp M. Rösler J. (2023). “Si-containing titanium alloys for laser powder bed fusion (PBF-L),” in Proceedings of the 61st conference of metallurgists, COM 2022 (Cham, Switzerland: Springer), 343–354. 10.1007/978-3-031-17425-4_46

[B28] Haase F. Siemers C. Klinge L. Lu C. Lang P. Lederer S. (2020). Aluminum- and vanadium-free titanium alloys for medical applications. MATEC Web Conf. 321, 05008. 10.1051/matecconf/202032105008

[B29] Haase F. Siemers C. Rösler J. (2022). Two novel titanium alloys for medical applications: thermo-mechanical treatment, mechanical properties, and fracture analysis. J. Mat. Res. 37, 2589–2603. 10.1557/s43578-022-00605-2

[B30] He J.-C. Zhu S.-P. Luo C. Niu X. Wang Q. (2022). Size effect in fatigue modelling of defective materials: application of the calibrated weakest-link theory. Int. J. Fatigue 165, 107213. 10.1016/j.ijfatigue.2022.107213

[B31] Kennedy J. R. Davis A. E. Caballero A. E. Byres N. Williams S. Pickering E. J. (2022). β Grain refinement by yttrium addition in Ti-6Al-4V wire-arc additive manufacturing. J. Alloy. Compd. 895, 162735. 10.1016/j.jallcom.2021.162735

[B32] Kennedy J. R. Davis A. E. Caballero A. E. Pickering E. J. Prangnell P. B. (2023). β grain refinement during solidification of Ti-6Al-4V in wire-arc additive manufacturing (WAAM). IOP Conf. Ser. Mat. Sci. Eng. 1274, 012005. 10.1088/1757-899X/1274/1/012005

[B33] LECO Corporation (2018). AMH43 software version 1.96. St. Joseph, MI: LECO Corporation.

[B34] Li Y. Yang C. Zhao H. Qu S. Li X. Li Y. (2014). New developments of Ti-based alloys for biomedical applications. Materials 7, 1709–1800. 10.3390/ma7031709 28788539 PMC5453259

[B35] Li Z. Zhang C. Qi L. Sun X. (2013). Selective laser melting bone-compatible pure titanium porous structure. Appl. Mech. Mat. 423-426, 833–836. 10.4028/www.scientific.net/AMM.423-426.833

[B36] Lütjering G. Williams J. C. (2007). Titanium. 2 edn. Berlin: Springer. 10.1007/978-3-540-73036-1

[B37] Málek J. Hnilica F. Veselý J. Smola B. Březina V. Kolařík K. (2014). The effect of boron addition on microstructure and mechanical properties of biomedical Ti35Nb6Ta alloy. Mat. Charact. 96, 166–176. 10.1016/j.matchar.2014.07.015

[B38] Mantri S. A. Alam T. Choudhuri D. Yannetta C. J. Mikler C. V. Collins P. C. (2017). The effect of boron on the grain size and texture in additively manufactured β-Ti alloys. J. Mat. Sci. 52, 12455–12466. 10.1007/s10853-017-1371-4

[B39] Murray J. L. (1986). “Ti-Y (titanium-yttrium),” in Binary alloy phase diagrams. Editor Massalski T. B. (Metals Park, Ohio: American Society for Metals), 2138.

[B40] Na T.-W. Kim W. R. Yang S.-M. Kwon O. Park J. M. Kim G.-H. (2018). Effect of laser power on oxygen and nitrogen concentration of commercially pure titanium manufactured by selective laser melting. Mat. Charact. 143, 110–117. 10.1016/j.matchar.2018.03.003

[B41] Nordin M. C. Edwards G. R. Olson D. L. (1987). The influence of yttrium microadditions on titanium weld metal cracking susceptibility and grain morphology. Weld. J. 34, 342s–352s.

[B42] Olympus Corporation (2011). Stream motion version 1.7. Olympus Corporation.

[B43] OriginLab Corporation (2023). OriginPro version 2023. Northampton, MA, USA: OriginLab Corporation.

[B44] Perevoshchikova N. Rigaud J. Sha Y. Heilmaier M. Finnin B. Labelle E. (2017). Optimisation of selective laser melting parameters for the Ni-based superalloy IN-738 LC using Doehlert’s design. Rapid Prototyp. J. 23, 881–892. 10.1108/RPJ-04-2016-0063

[B45] Schindelin J. Arganda-Carreras I. Frise E. Kaynig V. Longair M. Pietzsch T. (2012). Fiji: an open-source platform for biological-image analysis. Nat. Methods 9, 676–682. 10.1038/nmeth.2019 22743772 PMC3855844

[B46] Siemers C. Jencus P. Bäker M. Rösler J. Feyerabend F. (2007). “A new free machining titanium alloy containing lanthanum,” in Ti-2007. Science and Technology. Proceedings of the 11th world conference on titanium (JIMIC5), held at kyoto international conference center. Editors Niinomi M. Akiyama S. Ikeda M. Hagiwara M. Maruyama K. (Ichiban-cho, Aoba-ku, Sendai, Japan: The Japan Institute of Metals), 709–712.

[B47] Singh G. Ramamurty U. (2021). Reprint: boron modified titanium alloys. Prog. Mat. Sci. 120, 100815. 10.1016/j.pmatsci.2021.100815

[B48] Spieß L. Teichert G. Schwarzer R. Behnken H. Genzel C. (2019). Moderne Röntgenbeugung: Röntgendiffraktometrie für Materialwissenschaftler, Physiker und Chemiker 3 edn. Wiesbaden: Springer Spektrum. 10.1007/978-3-8348-8232-5

[B49] StJohn D. H. McDonald S. D. Bermingham M. J. Mereddy S. Prasad A. Dargusch M. (2018). The challenges associated with the formation of equiaxed grains during additive manufacturing of titanium alloys. Key Eng. Mat. 770, 155–164. 10.4028/www.scientific.net/KEM.770.155

[B50] Tamirisakandala S. Bhat R. B. Tiley J. S. Miracle D. B. (2005). Grain refinement of cast titanium alloys via trace boron addition. Scr. Mat. 53, 1421–1426. 10.1016/j.scriptamat.2005.08.020

[B51] Tamirisakandala S. Miracle D. B. (2010). Microstructure engineering of titanium alloys via small boron additions. Int. J. Adv. Eng. Sci. Appl. Math. 2, 168–180. 10.1007/s12572-011-0033-z

[B52] The MathWorks, Inc (2022a). MATLAB version 9.12 (R2022a). Natick, MA: The MathWorks, Inc.

[B53] The MathWorks, Inc (2022b). Statistics and machine learning Toolbox documentation, for MATLAB version R2022a. Natick, MA: The MathWorks, Inc.

[B54] The MathWorks, Inc (2022c). Statistics and machine learning Toolbox version 12.3 (R2022a). Natick, MA: The MathWorks, Inc.

[B55] Thijs L. Verhaeghe F. Craeghs T. Van Humbeeck J. Kruth J.-P. (2010). A study of the microstructural evolution during selective laser melting of Ti–6Al–4V. Acta Mater. 58, 3303–3312. 10.1016/j.actamat.2010.02.004

[B56] Tian Y. S. Chen C. Z. Chen L. X. Huo Q. H. (2006). Effect of RE oxides on the microstructure of the coatings fabricated on titanium alloys by laser alloying technique. Scr. Mat. 54, 847–852. 10.1016/j.scriptamat.2005.11.011

[B57] Toby B. H. (2005). CMPR – A powder diffraction toolkit. J. Appl. Cryst. 38, 1040–1041. 10.1107/S0021889805030232

[B58] Ueda K. Nakaoka S. Narushima T. (2013). β-Grain Refinement of α+β-Type Ti–4.5Al–6Nb–2Fe–2Mo Alloy by Using Rare-Earth-Oxide Precipitates. Mat. Trans. 54, 161–168. 10.2320/matertrans.MC201207

[B59] Wang D. W. Zhou Y. H. Shen J. Liu Y. Li D. F. Zhou Q. (2019). Selective laser melting under the reactive atmosphere: A convenient and efficient approach to fabricate ultrahigh strength commercially pure titanium without sacrificing ductility. Mat. Sci. Eng. A 762, 138078. 10.1016/j.msea.2019.138078

[B60] Wang X. Zhang L.-J. Ning J. Li S. Zhang L.-L. Long J. (2021). Hierarchical grain refinement during the laser additive manufacturing of Ti-6Al-4V alloys by the addition of micron-sized refractory particles. Addit. Manuf. 45, 102045. 10.1016/j.addma.2021.102045

[B61] Weng W. Biesiekierski A. Lin J. Ozan S. Li Y. Wen C. (2020). Impact of the rare earth elements scandium and yttrium on beta-type Ti-24Nb-38Zr-2Mo-base alloys for orthopedic applications. Materialia 9, 100586. 10.1016/j.mtla.2020.100586

[B62] Wysocki B. Maj P. Krawczyńska A. Rożniatowski K. Zdunek J. Kurzydłowski K. J. (2017). Microstructure and mechanical properties investigation of CP titanium processed by selective laser melting (SLM). J. Mat. Process. Technol. 241, 13–23. 10.1016/j.jmatprotec.2016.10.022

[B63] Xu P. Pyczak F. Yan M. Kong F. Ebel T. (2020). Impacts of yttrium on microstructure and tensile properties of biomedical β Ti-Nb-Zr fabricated by metal injection molding. Mat. Sci. Eng. A 792, 139816. 10.1016/j.msea.2020.139816

[B64] Xu W. Brandt M. Sun S. Elambasseril J. Liu Q. Latham K. (2015). Additive manufacturing of strong and ductile Ti–6Al–4V by selective laser melting via *in situ* martensite decomposition. Acta Mater. 85, 74–84. 10.1016/j.actamat.2014.11.028

[B65] Xue A. Lin X. Wang L. Wang J. Huang W. (2019). Influence of trace boron addition on microstructure, tensile properties and their anisotropy of Ti6Al4V fabricated by laser directed energy deposition. Mat. Des. 181, 107943. 10.1016/j.matdes.2019.107943

[B66] Zhang D. Prasad A. Bermingham M. J. Todaro C. J. Benoit M. J. Patel M. N. (2020a). Grain refinement of alloys in fusion-based additive manufacturing processes. Metall. Mat. Trans. A 51, 4341–4359. 10.1007/s11661-020-05880-4

[B67] Zhang D. Qiu D. Gibson M. A. Zheng Y. Fraser H. L. Prasad A. (2020b). Refining prior-β grains of Ti–6Al–4V alloy through yttrium addition. J. Alloy. Compd. 841, 155733. 10.1016/j.jallcom.2020.155733

[B68] Zhang K. Tian X. Bermingham M. Rao J. Jia Q. Zhu Y. (2019). Effects of boron addition on microstructures and mechanical properties of Ti-6Al-4V manufactured by direct laser deposition. Mat. Des. 184, 108191. 10.1016/j.matdes.2019.108191

[B69] Zhang L.-C. Chen L.-Y. Zhou S. Luo Z. (2023). Powder bed fusion manufacturing of beta-type titanium alloys for biomedical implant applications: A review. J. Alloy. Compd. 936, 168099. 10.1016/j.jallcom.2022.168099

[B70] Zhang L.-C. Chen L.-Y. (2019). A review on biomedical titanium alloys: recent progress and prospect. Adv. Eng. Mat. 21, 1801215. 10.1002/adem.201801215

[B71] ZwickRoell GmbH & Co. (2007). testXpert® II, version 3.0. Ulm, Germany: Zwick GmbH & Co.

[B72] ZwickRoell GmbH & Co. KG (n.d.). testXpert® III, version 1.51. Ulm, Germany: ZwickRoell GmbH & Co. KG.

